# Gene networks orchestrated by *MeGI*: a single‐factor mechanism underlying sex determination in persimmon

**DOI:** 10.1111/tpj.14202

**Published:** 2019-02-14

**Authors:** Ho‐Wen Yang, Takashi Akagi, Taiji Kawakatsu, Ryutaro Tao

**Affiliations:** ^1^ Graduate School of Agriculture Kyoto University Kyoto 606‐8502 Japan; ^2^ Japan Science and Technology Agency (JST) PRESTO Kawaguchi‐shi Saitama 332‐0012 Japan; ^3^ Division of Biotechnology Institute of Agrobiological Sciences National Agriculture and Food Research Organization Tsukuba Ibaraki 305‐8602 Japan

**Keywords:** ABC model, cistrome, dioecy, diospyros, evolution, gene network, sex determination, unisexual flower

## Abstract

Separating male and female sex organs is one of the main strategies used to maintain genetic diversity within a species. However, the genetic determinants and their regulatory mechanisms have been identified in only a few species. In dioecious persimmons, the homeodomain transcription factor, *MeGI*, which is the target of a Y chromosome‐encoded small‐RNA,*OGI*, can determine floral sexuality. The basic features of this system are conserved in the monoecious hexaploid Oriental persimmon, in which an additional epigenetic regulation of *MeGI* determines floral sexuality. The downstream regulatory pathways of *MeGI* remain uncharacterized. In this study, we examined transcriptomic data for male and female flowers from monoecious persimmon cultivars to unveil the gene networks orchestrated by *MeGI*. A network visualization and cistrome assessment suggested that class‐1 *KNOTTED‐like homeobox* (*KNOX*)/ovate family protein (OFP)/growth regulating factors (*GRF*s) and short vegetative phase (SVP) genes mediate the differences in gynoecium and androecium development between male and female flowers, respectively. The expression of these genes is directly controlled by *MeGI*. The gene networks also suggested that some cytokinin, auxin, and gibberellin signaling genes function cooperatively in the *KNOX/OFP/GRF* pathway during gynoecium differentiation. Meanwhile, *SVP* may repress *PI* expression in developing androecia. Overall, our results suggest that *MeGI* evolved the ability to promote gynoecium development and suppress androecium development by regulating *KNOX/OFP/GRF* and *SVP* expression levels, respectively. These insights may help to clarify the molecular mechanism underlying the production of unisexual flowers, while also elucidating the physiological background enabling a single‐factor system to establish dioecy in plants.

## Significance Statement

The evolution of sex determination systems has been a major focus in plant biology for a long time, whereas the genetic determinants or molecular pathways have not been well characterized. Transcriptomic analyses on developing flowers with various persimmon accessions unveiled the gene networks orchestrated by a sex‐determining gene, *MeGI*, which provided the insight into physiological mechanisms not only to develop unisexual flowers, but also to enable a ‘single‐factor’ system to evolve dioecy.

## Introduction

Sexuality is the main strategy used to maintain genetic diversity within a species. By contrast with animals, most angiosperms are hermaphroditic, which is the ancestral state of flowering plants. However, some angiosperms have evolved as monoecious species, in which each individual produces distinct male and female flowers, or as dioecious species, which produce male and female flowers on different individuals (Renner, 2014). Dioecy is thought to be often associated with the presence of sex chromosomes, which include genetic determinants of sex, although only a small part of the whole dioecious species have been assessed so far (Ming *et al*., 2011; Renner, 2014; Charlesworth, 2016). Recent advances in genomics‐based research have helped to reveal the structure of sex chromosomes in some dioecious plants (Liu *et al*., 2004; Ming *et al*., 2011; Wang *et al*., 2012; Charlesworth, 2016; Kazama *et al*., 2016; Harkess *et al*., 2017; Muyle *et al*., 2017). Additionally, a few genetic determinants of sex have been detected on sex chromosomes in some species, including persimmons (*Diospyros* spp.) (Akagi *et al*., 2014), garden asparagus (*Asparagus officinalis* L.) (Harkess *et al*., 2017), and kiwifruit (*Actinidia* spp.) (Akagi *et al*., 2018). The recent discovery of two Y chromosome‐encoded sex‐determining genes in garden asparagus, *aspTDF1* as the male‐promoting factor (M) and *SOFF* as the female‐suppressing factor (SuF), directly supports the ‘two‐mutation model’, which is a representative framework for the evolution of dioecy (Charlesworth and Charlesworth, 1978). The expression of a Y chromosome‐encoded sex‐determining gene identified in kiwifruit (Akagi *et al*., 2018), *Shy Girl*, can suppress female functions, which is also consistent with the two‐mutation model. On the other hand, in persimmons, a single gene located on the Y chromosome might be sufficient for determining sexuality. The *OGI* gene on the Y chromosome is a non‐coding RNA gene that produces a small‐RNA, and is a genetic determinant of sex in persimmons, while its autosomal counterpart, *MeGI*, is targeted by the *OGI* small‐RNA, and is thought to be the integrator of sex expression (Akagi *et al*., 2014). As Oriental persimmon (*Diospyros kaki*) evolved into a hexaploid species, its dioecious sex determination system was transferred into a more plastic system, including monoecious individuals, which are genetically male because they carry a Y chromosome (Akagi *et al*., 2016; Henry *et al*., 2018). Although the fundamental regulatory pathways in the monoecious individuals are likely to be identical to those of diploid dioecious *Diospyros* species, *OGI* is substantially silenced by a SINE‐like insertion in the promoter region (Akagi *et al*., 2016). By contrast, the epigenetic conditions of the *MeGI* promoter region and the resulting *MeGI* expression level are sufficient for determining the sex of each flower on monoecious trees. This implies that *MeGI* is the single integrator of sexuality in persimmons (Henry *et al*., 2018). Nevertheless, the molecular pathways underlying this integration by *MeGI* that is essential for androecia and gynoecia development remain uncharacterized.

Regarding the factors affecting plant sex expression, phytohormones have traditionally been considered to play important roles, although the effects are likely to differ across plant species (Grant *et al*., 1994). In particular, cytokinin signals are thought to be important for gynoecium development, for which the responsible molecular mechanisms have been well characterized in hermaphroditic model plant species such as *Arabidopsis thaliana* (Marsch‐Martínez *et al*., 2012). The treatment of male flowers with exogenous cytokinins often induces the development of gynoecia in some dioecious or monoecious plants, such as wild grape (*Vitis amurensis*) (Wang *et al*., 2013), kiwifruit (*Actinidia* spp.) (Akagi *et al*., 2018), and Oriental persimmon (*D. kaki*) (Yonemori *et al*., 1993). Genes encoding regulators of sex expression have recently been gradually unveiled. In *Silene latifolia*, components of the *CLV‐WUS* and *CUC‐STM* pathways are reportedly upregulated in a bisexual mutant that was putatively derived from a SuF‐disrupted male plant, suggesting that the Y chromosome‐encoded SuF in this species can regulate these pathways during the repression of gynoecium development (Koizumi *et al*., 2010). Similar pathways are also likely to affect gynoecium development in kiwifruit (Akagi *et al*., 2018). In garden asparagus, *AMS*,* MS2*,* LAP3* and *LAP5*, which are genes involved in a late pollen development stage, are male‐biased genes whose expression can be influenced by *aspTDF1* (Harkess *et al*., 2015, 2017). Additionally, the ABCDE model is well known for its role in the specification of floral organs, in which B type genes, *APETALA3* (*AP3*) and *PISTILLATA* (*PI*), can specify androecia development along with a C type gene, *AGAMOUS* (*AG*), and E type *SEP* genes (Yanofsky *et al*., 1990; Bowman *et al*., 1991; Weigel and Meyerowitz, 1994; Mizukami and Ma, 1997; Rijpkema *et al*., 2010). During early flower development in *A. thaliana*, short vegetative phase (SVP) and suppressor of overexpression of constans1 (*SOC1*), which were originally identified as flowering time‐related genes, may encode repressors of class B genes, *AP3* and *PI*, and a class C gene, *AG* (Wagner, 2008; Gregis *et al*., 2009). Differential B and C class gene expression levels are reportedly associated with the production of unisexual flowers in dioecious *Spinacia oleracea* and monoecious *Quercus suber* L. (Pfent *et al*., 2005; Sobral and Costa, 2017).

In this study, we aimed to identify the gene networks involved in the *MeGI*‐mediated differentiation of female and male flowers by analyzing transcriptomic data sets from various developing flowers in diverse monoecious *D. kaki* cultivars. Co‐expression networks have recently been commonly applied to integrate the information in large transcriptional data sets (Li *et al*., 2015; Liseron‐Monfils and Ware, 2015). The ‘guide‐gene approach’ (Serin *et al*., 2016) represents one of the effective strategies for co‐expression analyses, especially when key components of a specific pathway have been identified (Itkin *et al*., 2013; Serin *et al*., 2016). In this study, we used *MeGI* as the guide gene (or ‘bait gene’) to analyze the co‐expression network. We also revealed the candidate gene networks directly controlled by *MeGI*, in which two independent core paths regulate gynoecium and androecium development. The data presented herein may be useful for elucidating the molecular mechanisms underlying the production of unisexual flowers, while also clarifying the physiological background that enables a single‐gene system to establish dioecy.

## Results

### Transcriptome profiles in developing flowers

**Figure 1:**
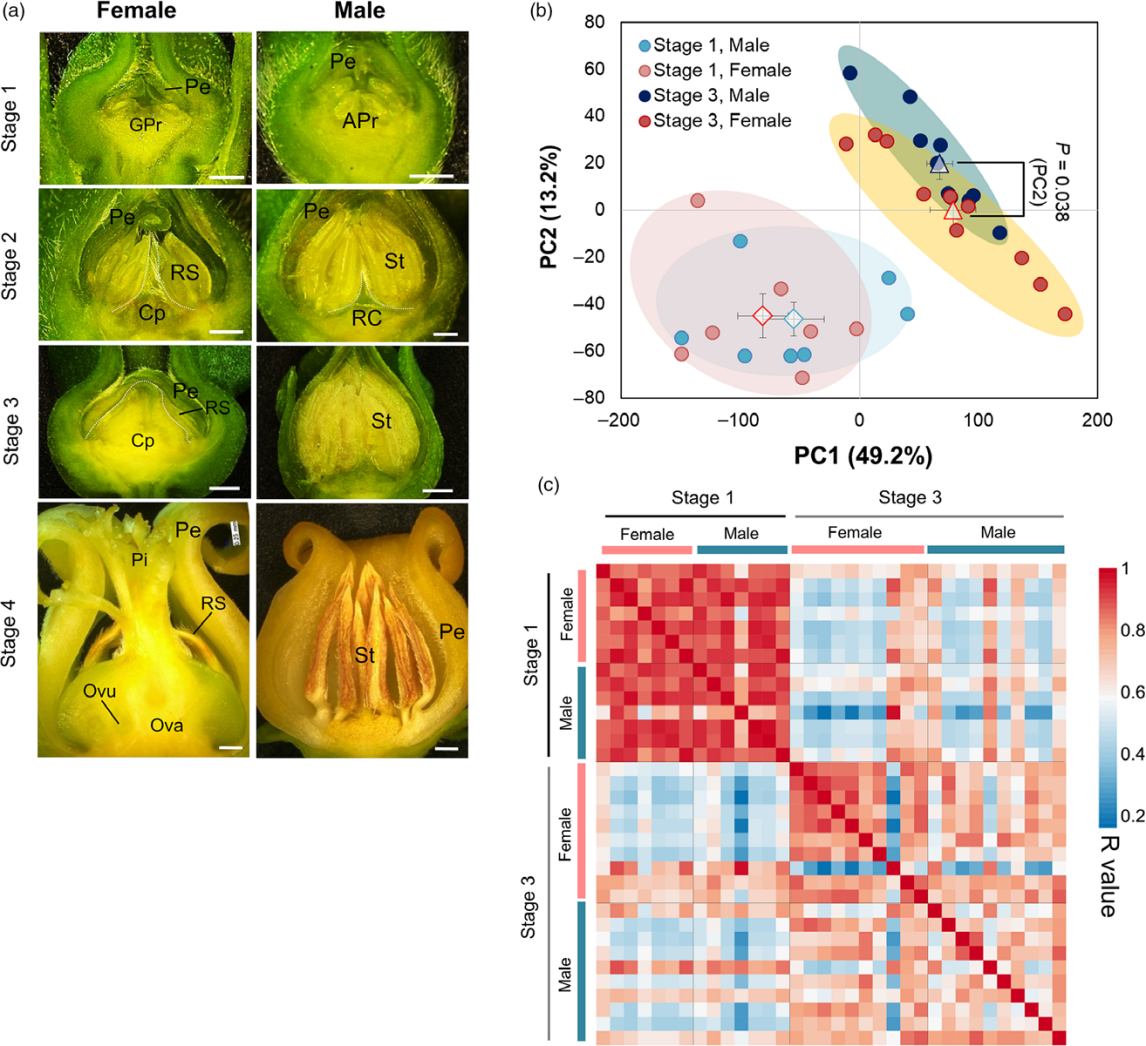
Transcriptomic profiles during male and female flower development. (a) Morphological observation and definition of the developmental stages of male and female flowers. At, anther; APr, androecium primordia; Cp, carpel; GPr, gynoecium primordia; Ova, ovary; Ovu, ovule; Pe, petal; Pi, pistil; RC, rudimentary carpel; RS, rudimentary stamen; St, stamen. Scale bars: 0.5 mm for stages 1 and 2, and 1.0 mm for stages 3 and 4. (b) Characterization of gene expression dynamics in male and female flowers by a PCA of expression data for each cultivar in stages 1 and 3. Male and female flowers were not substantially separated in stage 1, while significant differences were observed for the second component in stage 3. (c) Pearson correlation matrix of the gene expression data sets for 17 male and female flower samples collected in stages 1 and 3. For the PCA (b) and Pearson correlation analysis (c), normalized expression data for 15 644 genes with RPKM > 1 were used.

The developmental stages of *D. kaki* androecia/gynoecia from primordia initiation to maturation were morphologically divided into four stages (Figures 1a and S1). During these development stages, *MeGI* expression was substantially repressed by the methylation of the *MeGI* promoter and the accumulation of small RNA, which occurred in a male‐specific manner (Akagi *et al*., 2016). We sequenced the mRNA‐Seq Illumina libraries of each male and female flower collected in stage 1 for seven cultivars, and in stage 3 for 10 cultivars (Table S1). The reads were mapped onto the reference gene sequences of the diploid Caucasian persimmon, *Diospyros lotus* (Dlo_r1.0, http://persimmon.kazusa.or.jp/index.html), to calculate the expression levels as reads per kilobase of transcript per million mapped reads (RPKM). A principle component analysis (PCA) was conducted to profile the expression patterns of all genes that were substantially expressed (RPKM > 1.0) from stage 1 to stage 3 in male and female flowers (Figure 1b). PC1 and PC2 represented 42.9 and 13.2% of the total variance, respectively. The PCA clearly separated stages 1 and 3. Additionally, there were no significant differences in PC1 between female and male flowers in each stage, while significant differences were observed in PC2 between the male and female flowers in stage 3 (*P *=* *0.038). To further investigate the relationships between male and female flowers, Pearson's distance matrix was examined. The matrix revealed a strong correlation (*r *=* *0.87 in average) between female and male flowers in each cultivar (*N *=* *7) in stage 1, regardless of the genotype (Figure 1c). However, the matrix indicated the correlation between female and male flowers in each cultivar (*N *=* *10) was weaker in stage 3 (*r *=* *0.68 in average) than in stage 1. These results suggested that dynamic changes in the transcriptomes of male and female flowers occurred during the transition from stage 1 to stage 3. This tendency was consistent with the morphological characterization, in which only slight differences between male and female organs were detected in stage 1, while there were considerable variations in stage 3 (Figure 1a).

### Differentially expressed genes in male and female flowers

To analyze sex‐biased gene expression, some of which might be closely associated with *MeGI* expression, we attempted to identify the differentially expressed genes (DEGs) between female and male flowers in stages 1 and 3. We identified 1115 and 4720 DEGs [RPKM > 1, *P *<* *0.1; edgeR test with paired option (biological replicates *N *=* *7 × 2 and 10 × 2 for stage 1 and 3, respectively, see Materials and Methods)], and assigned putative functions according to the annotated *A. thaliana* genome (TAIR10) (Dataset S1). To simplify the analysis, each persimmon gene was called based on the putative orthologous genes or functions annotated in the TAIR10 database. The persimmon gene IDs are provided in Dataset S1.

**Figure 2:**
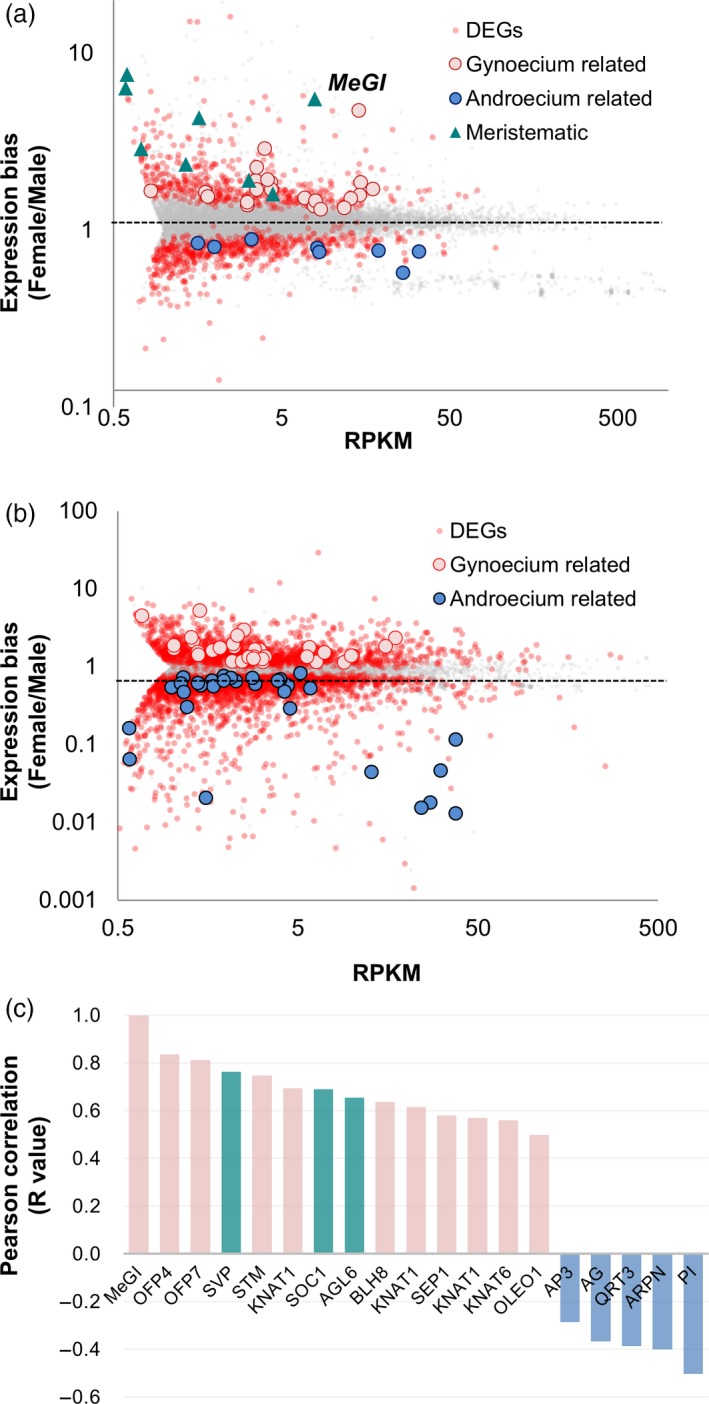
Detection of differentially expressed genes (DEGs) between male and female flowers, and correlations with the *MeGI* expression pattern.(a, b) Distribution of the expression patterns of the DEGs between male and female flowers in stage 1 (a) and stage 3 (b). The X and Y axes correspond to the normalized expression level (RPKM) and female/male expression ratio, respectively. The DEGs (*P *<* *0.01) are highlighted in red. The DEGs annotated with representative gynoecium‐related, androecium‐related, or (flowering‐related) meristematic functions are indicated with pink or blue circles or a green triangle, respectively. (c) Pearson correlation coefficients of functionally annotated DEGs against the *MeGI* expression pattern in stage 1 were calculated. Putative gynoecium‐related, androecium‐related, or meristematic genes are indicated with pink, blue, or green bars, respectively.

In stage 1, *MeGI* was identified as a female‐biased gene (Figure 2a). Moreover, genes related to meristem and gynoecium development were highly expressed in female flowers (Table S2a). For example, genes in the class‐1 *KNOTTED1‐like homeobox* (*KNOX*) subfamily, including shoot meristemless (STM), *BREVIPEDICELLUS/KNAT1* (*BP/KNAT1*), and *KNAT6*, which influence flower meristem and carpel development (Arnaud and Pautot, 2014), were more highly expressed in female flowers than in male flowers (Table S2a). Additionally, ovate family protein (OFP) genes, which affect ovule development, fruit shape, and secondary wall formation (Pagnussat *et al*., 2007; Tsaballa *et al*., 2011; Wang *et al*., 2011, 2016), were also biased toward female flowers. Meanwhile, some male‐biased genes reportedly influence stamen development. For example, the class B genes, *AP3* and *PI*, which are indispensable for androecium development (Weigel and Meyerowitz, 1994), were categorized as highly expressed male‐biased genes (Figure 2a, Table S2a). One of the class C genes, *AG*, which potentially contributes to the development of androecia and gynoecia (Bowman *et al*., 1991), was also identified as a male‐biased gene.

We expected to detect specific genes under the direct control of *MeGI* in stage 1, during which there were no morphological or dynamic gene expression differences between male and female flowers (Figure 1). Pearson's product‐moment correlation test between *MeGI* expression patterns and all transcripts revealed that some gynoecium‐related or meristematic‐related genes biased toward female flowers, such as *SVP*,* SOC1*,* AGL6*,* class‐1 KNOX*s and *OFPs*, were positively correlated with *MeGI* (Figure 2c, Table S2a). Conversely, genes biased toward male flowers exhibited a weaker correlation than the female‐biased genes (Figure 2c). One of the most negative correlations was observed between the expression levels of a representative androecium‐related gene, *PI* (*r *= −0.5).

In stage 3, we detected 4720 DEGs between female and male flowers (Figure 2b). Moreover, there was a substantial decrease in the *MeGI* expression level and no significant differences between male and female flowers (*P *>* *0.1). The 2212 female‐biased genes included representative genes related to gynoecium development (Figure 2b, Table S2b). Annotations of the 2508 male‐biased genes indicated pollen development, pollen wall assembly, and stamen development were enriched functions (Table S2b).

### Core gene networks correlated with *MeGI* expression

Next, we attempted to establish networks reflecting the relationships between the DEGs and *MeGI* by applying a weighted correlation network analysis (WGCNA) to identify the module (cluster) (Langfelder and Horvath, 2008) correlated with the *MeGI* expression pattern. To consider the genes expressed in developing flowers, we applied all transcriptomic data from stage 1 to stage 3 (*N *=* *44; Table S1) to construct co‐expression networks.

**Figure 3:**
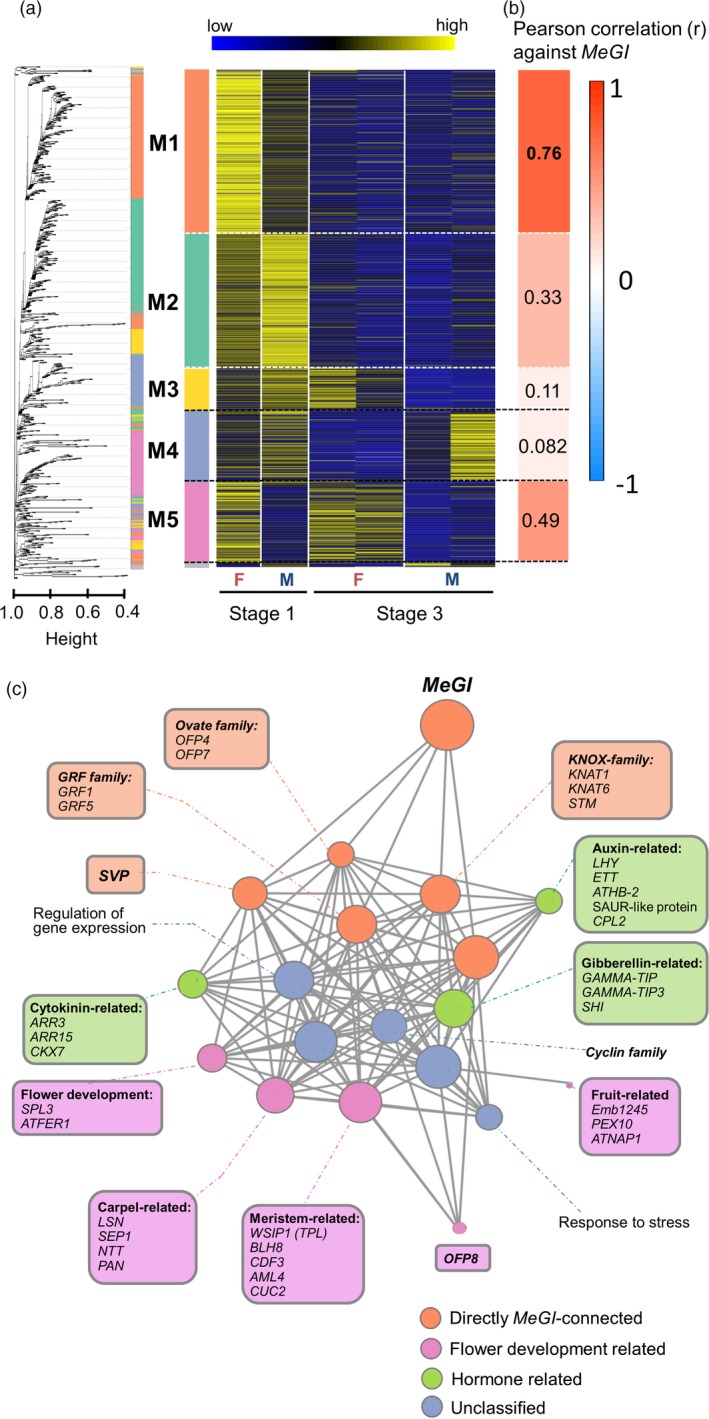
Clustering and networking of the DEGs in male and female flowers.(a) Clustering of the DEGs between male and female flowers in stage 1 with the WGCNA package revealed five main modules (M1–M5). For the heat map, 44 transcriptomic samples were divided into six categories: male (M) and female (F) flowers in stages 1 and 3, with an annual replication for stage 3. (b) Correlation coefficients of each module against the *MeGI* expression pattern were calculated with WGCNA. The M1 module (‘female module’), which includes *MeGI*, exhibited the highest correlation. (c) Visualization of the female module network. Genes are clustered according to their putative functions and are presented in different nodes. Additionally, *MeGI* and the first‐degree genes directly connected to *MeGI* are indicated in orange circles. The gene clusters putatively related to plant hormones and floral organ development are presented in green and pink circles, respectively. The genes annotated with other functions are indicated in blue (see Dataset S2). The size of the nodes reflect the number of edges connected to other nodes.

The DEGs between male and female flowers in stage 1 (*N *=* *1115) were first clustered into six modules, M1–M5 and ‘unclassified’ (Figure 3a,b). The M1 module included *MeGI* and 366 genes, which were mostly female‐biased DEGs (Dataset S2). Thus, we defined this module as the ‘female module’. Meanwhile, male‐biased genes were enriched in the M2 and M4 modules. Two genes likely involved in androecium formation, *PI* and *AG*, were nested in the M4 module, which we designated the ‘male module’ (Figure S2). A gene‐guide approach was used to assess the correlation between the expression levels of each module and *MeGI* (Serin *et al*., 2016). As expected, the highest correlation was observed between *MeGI* and the female module (*r *=* *0.76). A correlation between *MeGI* and the male module was detected (*r *=* *0.082) even though genes negatively correlated with *MeGI*, such as *AG* and *PI*, were included in this module. These results were likely due to the dilution of the specific genes significantly correlated with *MeGI* (Table S2, Dataset S2).

A co‐expression network analysis revealed that *MeGI* was directly connected to 18 of 366 genes in the female module. These 18 genes were divided into the following five groups: *SVP*, class‐1 *KNOX* family (*KNAT1*,* KNAT6*, and *STM*), *OFP* (*OFP7* and *OFP4*), growth regulating factors (GRFs), and unclassified genes (Figure 3c, Table S3). We set a topological overlap measure threshold for co‐expression at 0.17 based on the distribution of the number of edges. These 18 genes were defined as first‐degree genes directly connected to *MeGI* (Table S3). Consistent with our Pearson correlation test results (Figure 2c, Table S2), some meristematic‐related and/or gynoecium‐related genes, such as *SEPALLATA 1* (*SEP1*), were identified as second‐degree genes (Figure 3c). Genes related to fruit or embryo development, such as *PEROXISOME BIOGENESIS FACTOR 10* (*PEX10*) and *ARABIDOPSIS THALIANA NUCLEOSOME ASSEMBLY PROTEIN 1* (*ATNAP1*), were also detected in the female module, although they were not connected to *MeGI* (Dataset S2). Importantly, we identified genes biased toward the female flowers in stage 1 that were nested in the female module (Dataset S2), and were related to cytokinin, auxin, and gibberellin biosynthesis/signaling, including Arabidopsis response regulator 15 (ARR15) and cytokinin oxidase 7 (CKX7) (for cytokinin); carboxyl‐terminal domain (CTD), *PHOSPHATASE‐LIKE 2* (*CPL2*), late elongated hypocotyl (LHY), and *ETTIN* (*ETT*) (for auxin); and gamma tonoplast intrinsic protein (GAMMA‐TI*P*), gamma tonoplast intrinsic protein 3 (GAMMA‐TIP3), and short internodes (SHI) (for gibberellin) (Nemhauser *et al*., 2000; Fridborg *et al*., 2001; Hutchison and Kieber, 2002; Yanhui *et al*., 2006; Ueda *et al*., 2008; Köllmer *et al*., 2014).

The *SVP* gene, which encodes a regulator of the floral transition from vegetative to reproductive growth (Hartmann *et al*., 2000), was directly connected to *MeGI* in the female module (Figure 3c). Additionally, *SOC1* was included in the female module, but it was not directly connected to *MeGI*. As mentioned earlier in the manuscript, the *SVP* and *SOC1* genes mediate early flower development in *A. thaliana* by repressing class B genes (*AP3* and *PI*) and a class C gene (*AG*) (Wagner, 2008; Gregis *et al*., 2009), which affects androecium fertility. Although *SVP/SOC1* and *PI/AG* were nested in the female and male modules, respectively, the Pearson correlation matrix based on the expression level in stage 1 indicated that *SVP*,* SOC1*, and *MeGI* were negatively correlated with *AG* and *PI* (Figure S3). Consistent with this result, the pairwise correlation matrix for the female and male modules revealed a distinct negative correlation between these two modules (Figure S4), suggesting the modules may be functionally connected.

In the male module, in addition to *PI* and *AG*,* LIPOXYGENASE 4* (*LOX4*) and *SWEET7* may also affect androecium development (Bock *et al*., 2006, Rijpkema *et al*., 2010; Caldelari *et al*., 2011). Some genes related to auxin transport, which is critical for many aspects of androecium development (Cecchetti *et al*., 2008), were identified in the male module (Dataset S2), including auxin transporter protein 1 (AUX1) (Yang *et al*., 2006) and *BIG* (encoding a calossin‐like protein) (Gil *et al*., 2001). We also detected Arabidopsis response regulator 2 (ARR2), which is related to cytokinin signaling, and GAST1 protein homolog 1 (GASA1), which influences gibberellin signaling (Aubert *et al*., 1998; Borner *et al*., 2000) in the male module.

### Organ‐specificity in candidate gene expression

To examine the possibility that *MeGI* promotes gynoecium development and represses androecium development, RNA *in situ* hybridization was applied to localize *MeGI* expression. Substantial *MeGI* expression was observed in the center of the floral meristems of female flowers in stage 1 (Figure S5a), including both gynoecium and androecium primordia. However, we did not detect substantial *MeGI* expression in the androecium and gynoecium primordia of male flowers in stage 1 (Figure S5a), presumably because of gene silencing induced by DNA methylation and the accumulated small RNA (Akagi *et al*., 2016). Furthermore, we assessed the expression bias between the gynoecium and androecium regarding the genes putatively under the direct control of *MeGI* as described above. We also conducted an mRNA‐Seq analysis on developing gynoecia and (rudimentary) androecia in female flowers in stage 2. The expression of class‐1 *KNOX* and *OFP* genes was considerably biased toward the gynoecium, whereas *GRF* and *SVP* genes exhibited no bias toward either the androecium or gynoecium (Figure S5b). The *PI* expression level was significantly lower in female flowers than in male flowers as described, and was higher in the androecium than in the gynoecium of female flowers.

### Cistrome assessment to identify genes directly targeted by MeGI

**Figure 4:**
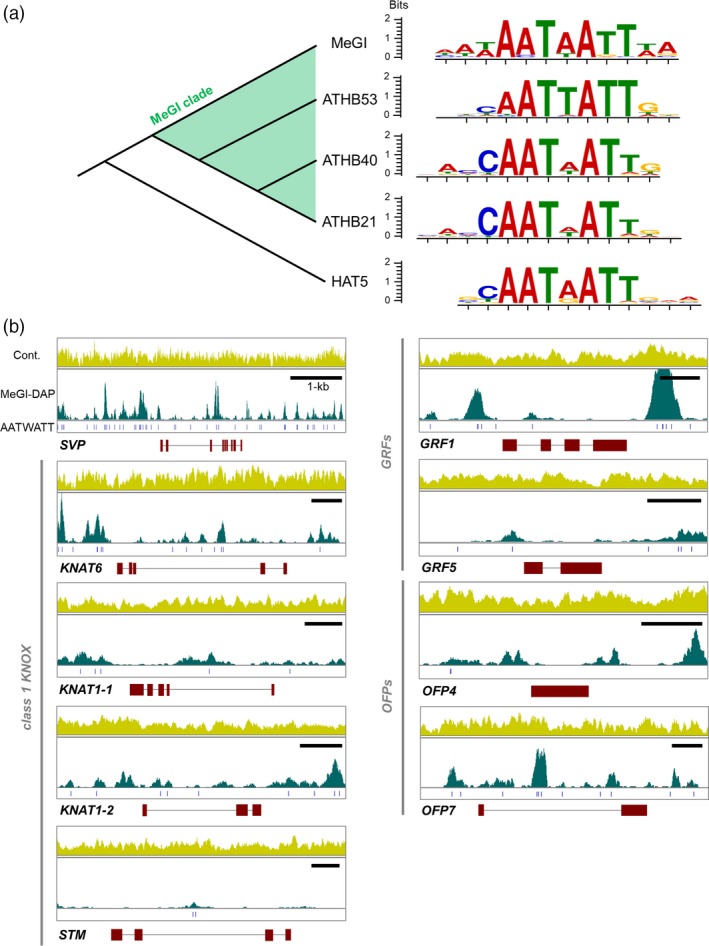
Cistrome analysis to identify the direct target motifs/genes of *MeGI*(a) Nucleotide motifs recognized by *MeGI* compared with the motifs bound by the other *Arabidopsis thaliana* HD‐ZIP1 transcription factors nested in the *MeGI* clade (Akagi *et al*., 2014). The enriched motifs were mostly identical for *MeGI* and three *MeGI* orthologs in *A. thaliana*, AtHB20, AtHB40, and AtHB53. (b) Ability of *MeGI* to bind to the genes directly connected to *MeGI* in the gene co‐expression network (see Figure 3c). The full‐length sequences (5′–3′) with introns and the 2‐kb regions 5′ upstream (left side) and 3′ downstream (right side) from the start and stop codons, respectively, were analyzed. For each gene, mapped read coverages in a random genomic DNA library (‘Cont’, yellow peaks) and in the DAP‐Seq library using a MeGI fusion protein (‘DAP‐MeGI’, green peaks), a MeGI‐recognizing motif (‘AATWATT’, blue bars), and gene structures (exons, red boxes) are provided. The DAP‐Seq peaks were almost consistent with the recognized motifs. Although MeGI could bind to the promoter sequences of all first‐degree genes, except for *STM*, the recognized motifs were especially enriched in *SVP*.

We applied DNA affinity purification sequencing (DAP‐Seq) (Bartlett *et al*., 2017) using MeGI fused to a Halo‐Tag to examine the genes and/or motifs directly targeted by MeGI. An Illumina gDNA library of the diploid Caucasian persimmon (*D. lotus*) cv. Kunsenshi‐male was filtered based on the affinity to the MeGI fusion protein. On the basis of DAP‐Seq data, we identified 72 746 MeGI‐ binding sites (peaks) *in vitro*. The DAP‐Seq reads were mapped to the *D. lotus* genome to characterize the accumulated recognition motifs by using MACS2 (Zhang *et al*., 2008). We also identified motifs using the top 2000 high‐confidence peaks, and determined that AATWATT was enriched in MeGI‐binding regions, by using MEME‐ChIP (Machanick and Bailey, 2011). This motif is similar to the binding motifs of closely related *A. thaliana* HD‐ZIPs, such as ATHB21, ATHB40, and ATHB53 (Figure 4a) (Khan *et al*., 2018). Among the genes directly connected to *MeGI* in the female module (Figure 3c), *SVP*,* KNOX* genes, and *OFP* genes were targeted by MeGI. Specifically, MeGI binds to the promoter regions and/or the introns of these genes at sites overlapping the AATWATT motif *in vitro* (Figure 4b). However, there was no significant peak detected in the promoter region and only one in the second intron of *STM*, which is also connected to *MeGI* in the female module (Figure 4b). These results suggest that MeGI can bind directly to the promoters of *SVP*,* KNOX* genes, and *OFP* genes to regulate their expression.

### Gene activation by ectopic expression of *MeGI* in *Arabidopsis thaliana*

**Figure 5:**
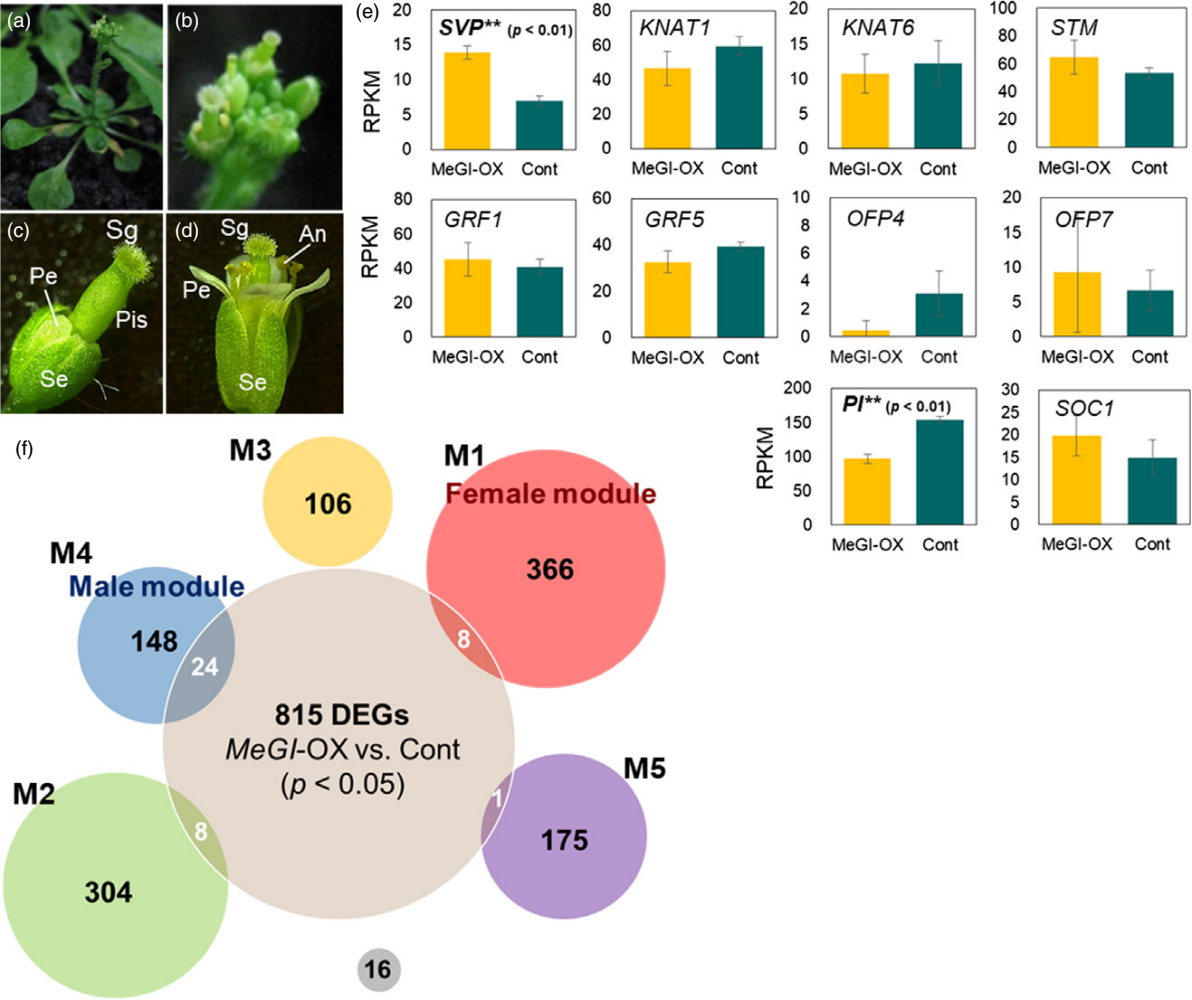
Transformation of *Arabidopsis thaliana* with CaMV35S‐*MeGI* to characterize the activated genes.(a–d) Representative phenotypes of the transgenic lines with CaMV35S‐*MeGI* (a–c) and the control plants (d). The *MeGI*‐overexpressing plants exhibited dwarfism (a) and repressed petal and anther development (b and c). An, anther; Pe, petal; Pis, pistil; Se, sepal; Sg, stigma. (e) Expression levels (RPKM) of the genes directly connected to *MeGI* in the co‐expression network (see Figure 3c) as well as *PI* and *SOC1*. Among these genes, only *SVP* and *PI* exhibited significant differences (*P *<* *0.01) between the *MeGI*‐overexpressing (MeGI‐OX) transgenic and control lines. Bars indicate standard errors. (f) Venn diagram of the DEGs detected between the MeGI‐OX and control *A. thaliana* plants (gray circle in the center, *N *=* *815), and between male and female persimmon flowers in stage 1. Genes were clustered into six modules (M1–M5 and unclassified, see Figure 3a). The male module (blue circle) had more overlapping orthologs with the *A. thaliana* DEGs than the other modules, which is consistent with the phenotypic changes in the MeGI‐OX lines, which mostly produced androecia. The *SVP* and *PI* genes were included in the overlapping areas for the female and male modules, respectively.

We previously proved that the overexpression of *MeGI* driven by the CaMV 35S promoter represses anther and petal development (Figure 5a–d). To investigate whether MeGI regulates the expression of conserved target genes in persimmon and *A. thaliana*, we completed an mRNA‐Seq analysis of CaMV35S‐*MeGI* and CaMV35S‐empty control lines (*N *=* *3 × 2 for biological replicates, Table S4) using developing flowers (or inflorescences) collected around stage 8 (Smyth *et al*., 1990). We observed that *MeGI* was specifically expressed in the CaMV35S‐*MeGI* lines (Table S4). Of the *A. thaliana* orthologs of the candidate target genes of *MeGI* (Figure 3c), the expression of *SVP* was significantly upregulated in the CaMV35S‐*MeGI* lines compared with the control lines (Figure 5e). However, *STM*, class‐1 *KNOX* genes (*KNAT1* and *KNAT6*), and *OFP* genes were not significantly affected by *MeGI* overexpression (Figure 5e, *P* > 0.1). This may have been due to the saturated expression of these genes because *A. thaliana* is originally hermaphroditic with a functional gynoecium that requires high class‐1 *KNOX* gene (e.g., STM and *KNAT1*) expression levels (Scofield *et al*., 2007) to develop. By contrast, *PI* expression was significantly downregulated in the CaMV35S‐*MeGI* lines compared with the control (Figure 5e). These results are consistent with our hypothetical path of *MeGI‐SVP‐PI*, and with the feminized phenotype of the transformed lines, similar to the *pi* mutant (Bowman *et al*., 1989; Figure 5a–d). We detected 815 DEGs (*P *<* *0.05) between the CaMV35S‐*MeGI* and control lines (Dataset S3). A comparison with the DEG orthologs in male and female persimmon flowers revealed that 16.2% (24/148) of the genes in the male module, including *PI*, overlapped with the CaMV35S‐*MeGI A. thaliana* DEGs (Figure 5f). Meanwhile, only 2.2% (8/366) of the genes in the female module, but including *SVP*, overlapped with the CaMV35S‐*MeGI A. thaliana* DEGs (Figure 5e, Dataset S3). These results were consistent with the observation that *SVP* expression is upregulated by *MeGI* in persimmon, resulting in the downregulated expression of *PI* and other male module genes. Of the 815 DEGs, most did no overlap with persimmon DEGs, presumably because *MeGI* was constitutively overexpressed in the CaMV35S‐*MeGI A. thaliana* lines, while *MeGI* expression in persimmon is limited to the meristematic region (Figure S5). However, although these DEGs were annotated with 12 statistically enriched GO terms (Table S5) (Tian *et al*., 2017), they were not directly related to the development or differentiation of the gynoecium/androecium.

## Discussion

In monoecious *D. kaki*, the DNA methylation pattern in the *MeGI* promoter, which affects *MeGI* expression directly or *via* the accumulated small‐RNA, determines floral sexuality (Akagi *et al*., 2016). Consequently, this species may be suitable for identifying the sex‐determination pathways controlled by *MeGI* during comparisons of male and female flowers from genetically identical individuals, similar to the comparative analysis of twins. Our transcriptomic data indicated *MeGI* was more highly expressed in female flowers than in male flowers, which is consistent with the results of previous studies (Akagi *et al*., 2014, 2016). Several floral organ identity genes, whose expression was synchronized with that of *MeGI*, were differentially expressed between female and male flowers. On the basis of a co‐expression network analysis and the identification of candidate target genes of *MeGI*, we were able to define two separate pathways governing androecium and gynoecium development.

Regarding androecium differentiation in male and female flowers, *SVP* (and possibly *SOC1*) encodes one of the main repressors of androecium development. Moreover, *SVP* expression was synchronized with that of *MeGI* in the male/female flowers of *D. kaki* and transgenic *A. thaliana* flowers overexpressing *MeGI*. Additionally, the *SVP* promoter region was directly targeted by *MeGI in vitro*. In *A. thaliana*, AP1 forms a dimer mainly with SVP (and/or SOC1) to repress the expression of class B and C genes during early flower developmental stages (Liu *et al*., 2008; Gregis *et al*., 2009). Consistent with this observation, our transcriptomic data for *D. kaki* and transgenic *A. thaliana* revealed a significantly negative correlation between *MeGI/SVP* and *PI* in the androecium. These results suggest that *SVP* can function as an important intermediate that connects the expression of *MeGI* and *PI* during androecium differentiation. Similar mechanisms likely exist in other dioecious species. Unisexual flowers have often been used to study the relationships between ABCDE‐like genes (Sather *et al*., 2010; Larue *et al*., 2013). For example, Sobral and Costa (2017) observed that in *Quercus suber* L., the class B genes, especially *QsPISTILLATA,* were predominantly expressed in male flowers, suggesting *PI* is mainly responsible for the sexual differentiation in developing androecia in diverse plant species.

Our results imply that during gynoecium differentiation in male and female flowers, the class‐1 *KNOX*,* OFP*, and/or *GRF* genes encode mediators that are directly controlled by *MeGI*. Their physiological functions may also be interrelated (Hackbusch *et al*., 2005), which is reminiscent of the signal‐mediated regulation of gibberellin and cytokinin (Frugis *et al*., 2001; Jasinski *et al*., 2005; Yanai *et al*., 2005). Cytokinin is essential for gynoecium development, from gynoecium initiation, embryonic development, and even fruit development (Müller and Sheen, 2007; Werner and Schmülling, 2009; Marsch‐Martínez *et al*., 2012). Female gametophyte arrest reportedly occurs in the Arabidopsis histidine kinase (AHP) mutants (*ahk2‐7 ahk3‐3 cre1‐12*) of *A. thaliana* (Cheng *et al*., 2013). Furthermore, a type‐C cytokinin response regulator gene in kiwifruit, *Shy Girl*, can serve as a Y chromosome‐encoded sex determinant, while the cytokinin signaling pathway genes are differentially expressed between male and female flowers (Akagi *et al*., 2018). Consistent with these observations, our results imply that the expression levels of three cytokinin metabolism/signaling genes (*ARR3*,* ARR15*, and *CKX7*) are positively correlated with *MeGI* and *KNOX*/*OFP*/*GRF* gene expression levels (Figure 3c). Furthermore, our co‐expression analysis confirmed that *STM* and *CUP‐SHAPED COTYLEDON 2* (*CUC2*), which activate cytokinin biosynthesis, are in the same female module. The cytokinin‐related pathway may be important for gynoecium differentiation in male and female persimmon flowers. This hypothesis is supported by the fact that treating male persimmon flowers with cytokinin leads to the production of hermaphroditic flowers (Yonemori *et al*., 1993).

Auxin and gibberellin signaling genes are also involved in gynoecium differentiation in persimmon, and function cooperatively with cytokinin signaling genes (Figure 3c, Table S2). The coordinated expression of auxin‐ and cytokinin‐related genes in the female module (Figure 3c) may be explained by crosstalk between the two plant hormones and can affect primordium formation and apical‐basal patterning during gynoecium development (Marsch‐Martínez *et al*., 2012; Besnard *et al*., 2014; Zuniga‐Mayo *et al*., 2014). However, auxin signaling is also essential for the late stages of stamen development. A lack of auxin signaling in the androecium causes precocious pollen maturation, anther dehiscence, and irregular filament development (Cecchetti *et al*., 2008). We observed that auxin‐related genes, such as *CPL2*,* LHY*, and *ETT*, were more highly expressed in male flowers than in female flowers in stage 3 (Dataset S1, Table S2). The gibberellin signal in the shoot apical meristem (SAM) is integrated by class‐1 *KNOX* genes (Sakamoto *et al*., 2001; Jasinski *et al*., 2005), and can repress cytokinin signaling (Greenboim‐Wainberg *et al*., 2005; Fleishon *et al*., 2011). Thus, among the female module genes, the lower expression levels of gibberellin‐related genes in female flowers than in male flowers (Dataset S2, Table S2) are consistent with our results described above. The expression of gibberellin‐regulated genes, such as *GASA1*, is also mediated by auxin (Paponov *et al*., 2008). Gibberellin signaling is often involved in activities associated with the formation of unisexual flowers, such as the abortion of androecium primordia in female flowers in maize (Lebel‐Hardenack and Grant, 1997) and the promotion of male flower development in cucumber (*Cucumis sativus* L.) (Zhang *et al*., 2017). Our results suggest that gibberellin signaling is important for sex determination in persimmon, potentially through cooperative effects with cytokinin signals.

**Figure 6:**
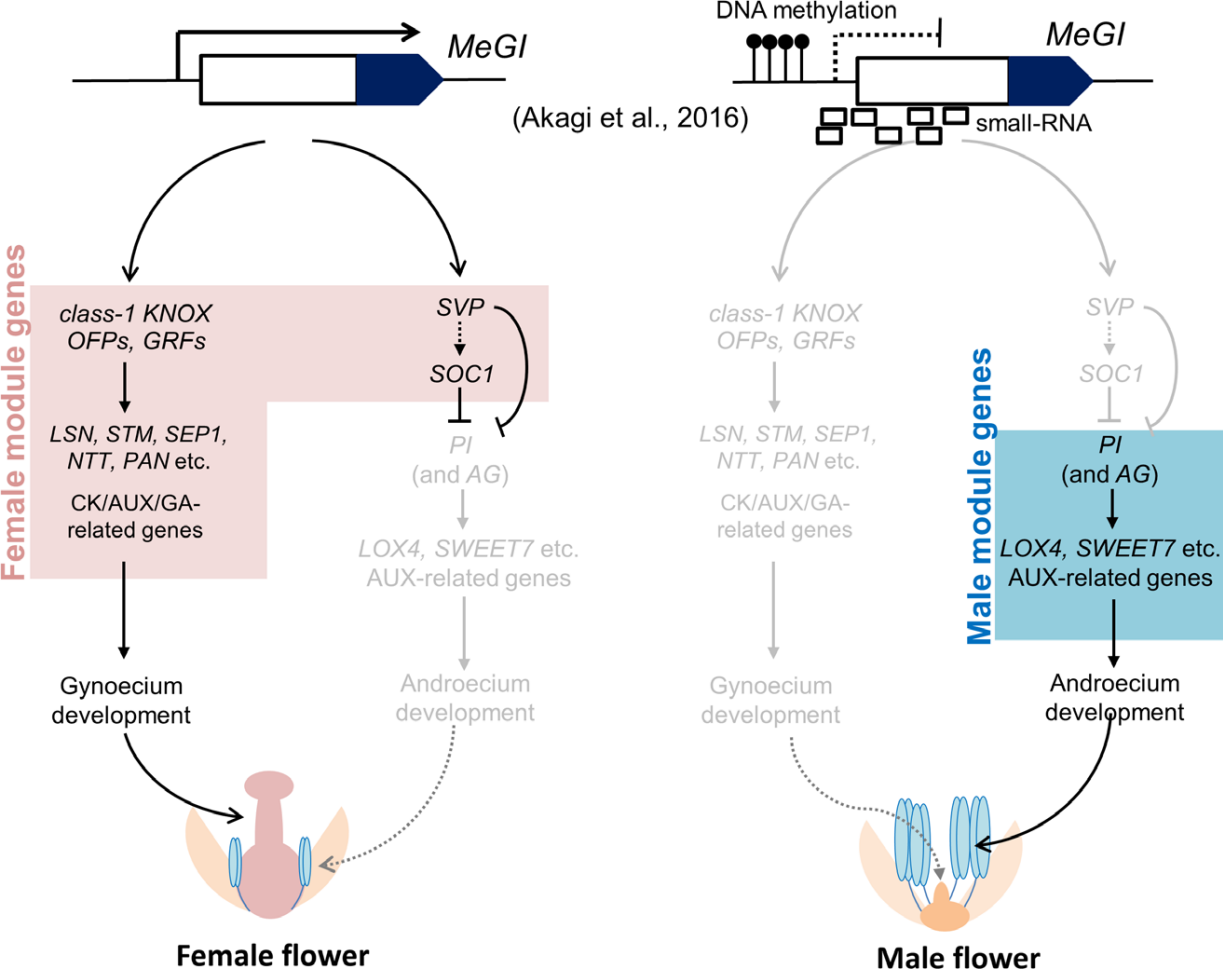
Model for the single‐factor sex determination mechanism of persimmon.*MeGI* can integrate two independent pathways to promote gynoecium development and repress androecium development. The class‐1 *KNOX*,* OFP*, and *GRF* genes can function as intermediates that activate cytokinin (CK)‐dependent pathways [and cooperative auxin (AUX)‐ and gibberellin (GA)‐related genes] to induce gynoecium development. However, *SVP* (and potentially *SOC1*) is important for repressing *PI* expression, thereby leading to an aborted androecium. In hexaploid *D. kaki*,* MeGI* expression is regulated by the epigenetic status of *MeGI* (Akagi *et al*., 2016). In diploid dioecious persimmon species, male individuals with a Y‐chromosome stably express *OGI*, resulting in an accumulation of small‐RNA targeting *MeGI*. In both cases, the *MeGI* expression level is sufficient for determining floral sexuality.

Although the two‐mutation model is a representative framework for the evolution of dioecy, a single‐factor model has been proposed for the dioecious sex determination system in persimmons (Akagi *et al*., 2014, 2016; Henry *et al*., 2018). However, the underlying physiological mechanism has not been characterized. The data presented herein may provide some physiological evidence for a single‐factor model, in which *MeGI* regulates two independent pathways for androecium and gynoecium development (Figure 6). We hypothesize that *MeGI‐SVP‐PI* and *MeGI‐KNOX/OFP/GRF* pathways are responsible for the differences in androecium and gynoecium differentiation between male and female flowers, respectively. Although the downstream genes or mechanisms in these core pathways must still be identified, we observed some commonalities in the regulation of gene/plant hormones among dioecious plants or unisexual flowers. Overall, the results of this study imply that persimmons evolved a sex determination system in which *MeGI* controls the separate androecium and gynoecium pathways, while the downstream mechanisms affecting the development of these organs may be similar in diverse plant species.

## Experimental procedures

### Plant materials

The developmental stages of the Oriental persimmon (*Diospyros kaki* Thunb.) buds/flowers were defined in previous studies (Yonemori *et al*., 1993; Akagi *et al*., 2016). In this study, we further divided the early flower development period into the following four stages based on observations of gynoecia and androecia development: stage 1 (before organ differentiation; 1–4 April 2016), stage 2 (organs are developing; 14–17 April 2016), stage 3 (organs are maturing; 28–30 April 2016, and 27–29 April 2015), and stage 4 (flowering; 10–15 May 2016). To construct mRNA‐Seq libraries, male and female flowers were separately harvested from seven monoecious cultivars in stage 1 (‘Tohachi’, ‘Okugosho’, ‘Amayotsumizo’, ‘Iwasedo’, ‘Egosho’, ‘Meotogaki’ and ‘Taiwanshoshi’), and from 10 monoecious cultivars in stage 3 (the seven cultivars mentioned before and ‘Taishu’, ‘Zenjimaru’, and ‘Kakiyamagaki’). Additionally, the pistil of female flowers and the stamen of male flowers were collected from ‘Taiwanshoshi’ plants in stage 2 for an organ‐specific mRNA‐Seq analysis.

### RNA isolation and Illumina sequencing

Total RNA was extracted from flower, pistil, and stamen samples using the PureLink^®^ Plant Reagent (Invitrogen, Carlsbad, CA, USA), after which the mRNA was isolated using the Dynabeads™ mRNA Purification Kit (Ambion, Foster City, CA, USA). Illumina sequencing libraries were prepared as previously described (Akagi *et al*., 2016, 2018). Briefly, cDNA was synthesized with random primers and Superscript III (Life Technologies, Carlsbad, CA, USA) followed by a heat inactivation for 5 min at 65°C. Second‐strand cDNA was synthesized in second‐strand buffer (200 mm Tris−HCl, pH 7.0, 22 mm MgCl_2_, and 425 mm KCl) containing DNA polymerase I (NEB, Ipswich, MA, USA) and RNase H (NEB). Samples were incubated at 16°C for 2.5 h. The resulting double‐stranded cDNA was used to prepare libraries with the KAPA Hyperplus Kit (Kapa Biosystems, Wilmington, MA, USA), while AMPureXP (Beckman Coulter Life Science, Brea, CA, USA) was used to remove fragments shorter than 300 bp. The purified libraries were quantified with a Qubit 2.0 fluorometer (Invitrogen) and then analyzed with the Illumina HiSeq 4000 system at the QB3 Genomic Sequencing Laboratory of UC Berkeley (http://qb3.berkeley.edu/gsl/). The resulting 50‐bp single‐end reads were analyzed at the Vincent J. Coates Genomics Sequencing Laboratory at UC Berkeley. Raw sequencing reads were processed using custom Python scripts developed in the Comai laboratory, which are available online (http://comailab.genomecenter.ucdavis.edu/index.php/Barcoded_data_preparation_tools), as previously described (Akagi *et al*., 2014).

### Transcriptomic data profiling

The mRNA‐Seq Illumina reads were aligned to the reference coding sequences (CDSs) of the diploid Caucasian persimmon, *D. lotus* (http://persimmon.kazusa.or.jp/index.html), using the default parameters of the Burrows‐Wheeler Aligner (version 0.7.12) (Li and Durbin, 2009, https://github.com/Ih3/bwa). The read counts per CDS were determined from the aligned SAM files using a custom R script to calculate the RPKM for each gene. To examine the gene expression dynamics in female and male flowers collected in stages 1 and 3, a PCA was conducted using prcomp in R. Additionally, Pearson's product‐moment correlation coefficients were calculated for each sample, using the cor.test with the ‘pearson’ argument in R, with gene expression levels as the parameters.

### Identification of differentially expressed genes

Genes that were differentially expressed between female and male flowers were detected with edgeR (Robinson *et al*., 2010; McCarthy *et al*., 2012), using the paired‐test option, an in‐house R script, as well as 7 and 10 biological replicates for stages 1 and 3, respectively. Male and female flowers from the same cultivars were paired and used as biological replicates. The DEGs were filtered according to RPKM and *P‐*values (RPKM ≥ 1.0, *P *<* *0.1). Putative functions of each gene were determined with a BLASTX search of the TAIR10 database (https://www.arabidopsis.org/index.jsp). A Pearson correlation analysis was also applied for determining the correlation between the expression levels of DEGs and *MeGI* in each stage, using the cor.test with the ‘pearson’ argument in R.

### Construction of the co‐expression network

The DEGs in stage 1 were selected to construct a gene co‐expression network based on the WGCNA package, which is a representative algorithm used for developing co‐expression networks (Langfelder and Horvath, 2008). The soft‐thresholding power for a signed network was set at 6, with a scale‐free model fitting index *R* ^2 ^> 0.8. A relatively large minimum module size (30) and a medium sensitivity (deepSplit = 2) to cluster splitting were also set. In the co‐expression network, genes were represented by nodes, and the correlation values (weight) between two genes were calculated by raising Pearson's correlation coefficient. The genes in the same module were first visualized with the VisANT program (Hu *et al*., 2004), and only lines with a weight greater than 0.175 and 0.065 were visualized in the module with *MeGI* and the module with genes related to the formation of male organs, respectively. The final networks were designed with the igraph and ggplot2 packages (Csardi and Nepusz, 2006; Wickham, 2009).

### DAP‐Seq analysis

The DAP genomic DNA library was prepared and the DAP reaction was completed as previously described (O'Malley *et al*., 2016; Bartlett *et al*., 2017). Briefly, the Covaris M220 ultrasonicator (with the manufacturer‐recommended setting) was used to fragment gDNA to an average size of 200 bp. The resulting fragmented gDNA was end‐repaired with the End‐It DNA Repair kit (Epicentre, Madison, WI, USA). Next, a dA‐tail was added using the Klenow fragment (3′→5′ exo‐) (NEB). The DAP‐Seq adaptor (i.e., truncated Illumina TruSeq adapter) was ligated to the fragmented gDNA with T4 DNA polymerase (NEB). Full‐length *MeGI* cDNA was cloned into the pDONR221 vector (Life Technologies) and then transferred to pIX‐Halo using LR clonase II (Life Technologies) to generate pIX‐Halo‐MeGi. The N‐terminally Halo‐tagged MeGI was produced using the TNT SP6 Coupled Wheat Germ Extract System (Promega, Fitchburg, WI, USA) and purified with Magne Halo‐Tag beads (Promega). In total, 50 ng DAP gDNA library was incubated with Halo‐tagged MeGI at room temperature for 1 h. The recovered library was sequenced with the Illumina HiSeq 4000 system at the Vincent J. Coates Genomics Sequencing Laboratory at UC Berkeley.

### RNA *in situ* hybridization

The *MeGI* cDNA was cloned into the pGEM‐T Easy vector (Promega). Additionally, RNA probes for the sense‐*MeGI* and antisense‐*MeGI* sequences were labeled with the DIG RNA Labeling Kit (SP6/T7) (Roche, Mannheim, Germany). The RNA *in situ* hybridization analysis was completed as described by Akagi *et al*. (2018), with minor modifications. Specifically, female and male flowers collected in stage 1 were fixed in FAA (1.8% formaldehyde, 5% acetic acid, and 50% ethanol). The FAA was then replaced by a 10–30% sucrose solution series before flower samples were sliced with a CM1520 cryostat (Leica, Wetzlar, Germany) using cryofilm as previously described (Kawamoto, 2003). The tissues were sliced into approximately 10‐μm sections, and mounted on Frontier coated glass slides (Matsunami Glass Ind., Kishiwada, Japan). The tissue sections were rehydrated in an ethanol series and then incubated in a Proteinase K solution (700 U ml^−1^ Proteinase K, 50 mm EDTA, 0.1 m Tris−HCl, pH 7.5) for 30 min at 37°C, followed by an acetylation with acetic anhydride (0.25% acetic anhydride in 0.1 m triethanolamine solution) for 10 min. The full‐length *SyGI* cDNA sequence was cloned into the pGEM‐T Easy vector (Promega) to synthesize DIG‐labeled antisense RNA probes using the DIG‐labeling RNA synthesis kit (Roche, Switzerland). The probe solution including RNaseOUT (Thermo Fisher Scientific, Waltham, MA, USA) was applied to the slides, which were then covered with Parafilm. Hybridizations were completed at 48°C for >16 h. For the subsequent detection, 0.1% anti‐digoxigenin‐AP Fab fragments (Sigma‐Aldrich, St. Louis, MO, USA) were used as the secondary antibody, which were visualized with NBT/BCIP solutions.

### Transformation and transcriptomic analysis of transgenic *Arabidopsis thaliana*

The full‐length *MeGI* sequence was inserted into the pPLV26 vector (Rybel *et al*., 2011) in an earlier study (Akagi *et al*., 2014). *A. thaliana* plants were transformed with *MeGI* as described by Akagi *et al*. (2018). Briefly, *A. thaliana* ecotype Columbia‐0 plants were grown at 21°C under white light (400–750 nm) with a 16‐h light/8‐h dark photoperiod. The pPLV26‐*MeGI* construct was introduced into *Agrobacterium tumefaciens* strain EHA105 by electroporation along with the helper vector pSOUP. Wild‐type *A. thaliana* plants were transformed using a floral‐dip method. The putative transgenic plants were screened on Murashige and Skoog medium containing 30 μg ml^−1^ kanamycin.

The mRNA‐Seq analysis was completed using developing flowers that were collected from three plants for each pPLV26‐MeGI transgenic and control line around stage 8 (Smyth *et al*., 1990). The mRNA‐Seq libraries were constructed and sequenced as described above. The Illumina reads were aligned to the reference CDSs of *A. thaliana* (Araport 11) (https://www.arabidopsis.org/download_files/Genes/Araport11_genome_release/Araport11_blastsets/Araport11_genes.201606.cds.fasta.gz) using the default parameters of the Burrows‐Wheeler Aligner. The read counts per CDS were calculated based on the aligned SAM files using a custom R script. Differences in gene expression levels between the pPLV26‐*MeGI* transgenic and control plants were detected with the edgeR package in R (version 3.0.1).

### Accession numbers

All sequence data generated in this study have been deposited in the appropriate DDBJ database, with the Illumina reads for the mRNA‐Seq analysis deposited in the Short Read Archive database (BioProject ID PRJDB7688).

## Author Contributions

T.A., and R.T. conceived the study. Y.H. and T.A. designed the experiments. Y.H., T.A. and T.K. conducted the experiments. Y.H. and T.A. analyzed the data. Y.H. and T.A. drafted the manuscript. All authors approved the manuscript.

## Conflict of Interest

The authors declare no conflicts of interest.

## Supporting information


**Figure S1**. Morphological characterization of stage 1 and stage 3.
**Figure S2**. Visualization of the male module network.
**Figure S3**. Pearson correlation matrix in the *MeGI‐SVP‐SOC1* and *PI/AG*.
**Figure S4**. Pearson correlation matrix in the whole genes between the female and male modules.
**Figure S5**. Organ‐specificity in expression of the *MeGI* and the downstream candidates.

**Table S1**. List of plant materials.
**Table S2**. List of the representative DEGs putatively related to androecium, gynoecium, and meristem development in stage 1 (a) and stage 3 (b).
**Table S3**. Genes directly connected to *MeGI* in the female module.
**Table S4**. Phenotypes of the *MeGI*‐overexpressed and control transgenic lines.
**Table S5**. Enriched GO terms in the DEGs between the *MeGI*‐overexpressed and control in Arabidopsis.

**Dataset S1**. Information of DEGs with persimmon IDs and annotation by TAIR.

**Dataset S2**. Genes list in each module.

**Dataset S3**. List of DEGs in the *MeGI*‐OX and control in Arabidopsis.

## References

[tpj14202-bib-0001] Akagi, T. , Henry, I.M. , Tao, R. and Comai, L. (2014) A Y‐chromosome‐encoded small RNA acts as a sex determinant in persimmons. Science, 346, 646–650. 10.1126/science.1257225. 25359977

[tpj14202-bib-0002] Akagi, T. , Henry, I.M. , Kawai, T. , Comai, L. and Tao, R. (2016) Epigenetic regulation of the sex determination gene *MeGI* in polyploid persimmon. Plant Cell, 28, 2905–2915. 10.1105/tpc.16.00532. 27956470 PMC5240738

[tpj14202-bib-0003] Akagi, T. , Henry, I.M. , Ohtani, H. , Beppu, K. , Kataoka, I. and Tao, R. (2018) A Y‐encoded suppressor of feminization arose via lineage‐specific duplication of a cytokinin response regulator in kiwifruit. Plant Cell, 30, 780–795. 10.1105/tpc.17.00787. 29626069 PMC5969274

[tpj14202-bib-0101] Arnaud, N. and Pautot, V. (2014) Ring the BELL and tie the KNOX: roles for TALEs in gynoecium development. Front Plant Sci., 5, 93 10.3389/fpls.2014.00093. 24688486 PMC3960571

[tpj14202-bib-0004] Aubert, D. , Chevillard, M. , Dorne, A.M. , Arlaud, G. and Herzog, M. (1998) Expression patterns of *GASA* genes in Arabidopsis thaliana: the *GASA4* gene is up‐regulated by gibberellins in meristematic regions. Plant Mol. Biol. 36, 871–883. 10.1023/A:1005938624418. 9520278

[tpj14202-bib-0005] Bartlett, A. , O'Malley, R.C. , Huang, S.C. , Galli, M. , Nery, J.R. , Gallavotti, A. and Ecker, J.R. (2017) Mapping genome‐wide transcription‐factor binding sites using DAP‐seq. Nat. Protoc., 12, 1659–1672. 10.1038/nprot.2017.055. 28726847 PMC5576341

[tpj14202-bib-0109] Besnard, F. , Refahi, Y. , Morin, V. ***et al.*** (2014) Cytokinin signalling inhibitory fields provide robustness to phyllotaxis. Nature, 505, 417–421. 10.1038/nature12791. 24336201

[tpj14202-bib-0103] Bock, K.W. , Honys, D. , Ward, J.M. , Padmanaban, S. , Nawrocki, E.P. , Hirschi, K.D. , Twell, D. and Sze, H. (2006) Integrating membrane transport with male gametophyte development and function through transcriptomics. Plant Physiol, 140, 1151–1168. 10.1104/pp.105.074708. 16607029 PMC1435806

[tpj14202-bib-0006] Borner, R. , Kampmann, G. , Chandler, J. , Gleißner, R. , Wisman, E. , Apel, K. and Melzer, S. (2000) A MADS domain gene involved in the transition to flowering in Arabidopsis. Plant J. 24, 591–599. 10.1046/j.1365-313x.2000.00906.x. 11123798

[tpj14202-bib-0007] Bowman, J.L. , Smyth, D.R. and Meyerowitz, E.M. (1989) Gene directing flower development in Arabidopsis. Plant Cell, 1, 37–52. 10.1105/tpc.1.1.37. 2535466 PMC159735

[tpj14202-bib-0008] Bowman, J.L. , Smyth, D.R. and Meyerowitz, E.M. (1991) Genetic interactions among floral homeotic genes of Arabidopsis. Development, 112, 1–20. 1685111 10.1242/dev.112.1.1

[tpj14202-bib-0009] Caldelari, D. , Wang, G. , Farmer, E.E. and Dong, E. (2011) Arabidopsis *lox3 lox4* double mutants are male sterile and defective in global proliferative arrest. Plant Mol. Biol. 75, 25–33. 10.1007/s11103-010-9701-9. 21052784

[tpj14202-bib-0104] Cecchetti, V. , Altamura, M.M. , Falasca, G. , Costantino, P. and Cardarelli, M. (2008) Auxin regulates *Arabidopsis anther dehiscence*, pollen maturation, and filament elongation. Plant Cell, 20, 1760–1774. 10.1105/tpc.107.057570. 18628351 PMC2518247

[tpj14202-bib-0010] Charlesworth, D. (2016) Plant sex chromosomes. Annu. Rev. Plant Biol. 67, 397–420. 10.1146/annurev-arplant-043015-111911. 26653795

[tpj14202-bib-0011] Charlesworth, B. and Charlesworth, D. (1978) A model for the evolution of dioecy and gynodioecy. Am. Nat. 112, 975–997. https://www.jstor.org/stable/2460344.

[tpj14202-bib-0012] Cheng, C.Y. , Mathews, D.E. , Schaller, G.E. and Kieber, J.J. (2013) Cytokinin‐dependent specification of the functional megaspore in the Arabidopsis female gametophyte. Plant J. 73, 929–940. 10.1111/tpj.12084. 23181607

[tpj14202-bib-0112] Csardi, G. and Nepusz, T. (2006) The igraph software package for complex network research, InterJournal, Complex Systems 1695. http://igraph.org.

[tpj14202-bib-0013] Fleishon, S. , Shani, E. , Ori, N. and Weiss, D. (2011) Negative reciprocal interactions between gibberellin and cytokinin in tomato. New Phytol. 190, 609–617. 10.1111/j.1469-8137.2010.03616.x. 21244434

[tpj14202-bib-0014] Fridborg, I. , Kuusk, S. , Robertson, M. and Sundberg, E. (2001) The Arabidopsis protein *SHI* represses gibberellin responses in Arabidopsis and barley. Plant Physiol. 127, 937–948. 10.1104/pp.010388. 11706176 PMC129265

[tpj14202-bib-0015] Frugis, G. , Giannino, D. , Mele, G. , Nicolodi, C. , Chiappetta, A. , Bitonti, M.B. , Innocenti, A.M. , Dewitte, W. , Van Onckelen, H. and Mariotti, D. (2001) Overexpression of *KNAT1* in lettuce shifts leaf determinate growth to a shoot‐like indeterminate growth associated with an accumulation of isopentenyl‐type cytokinins. Plant Physiol. 126, 1370–1380. 10.1104/pp.126.4.1370. 11500537 PMC117138

[tpj14202-bib-0106] Gil, P. , Dewey, E. , Friml, J. , Zhao, Y. , Snowden, K.C. , Putterill, J. , Palme, K. , Estelle, M. and Chory, J. (2001) BIG: a calossin‐like protein required for polar auxin transport in Arabidopsis. Gene Dev., 15, 1985–1997. http://www.genesdev.org/cgi/doi/10.1101/gad.905201. 11485992 10.1101/gad.905201 PMC312751

[tpj14202-bib-0016] Grant, S. , Houben, A. , Vyskot, B. , Siroky, J. , Pan, W.H. , Macas, J. and Saedler, H. (1994) Genetics of sex determination in flowering plants. Genet. Dev. 15, 214–230. 10.1016/S1360-1385(97)01012-1.

[tpj14202-bib-0017] Greenboim‐Wainberg, Y. , Maymon, I. , Borochov, R. , Alvarez, J. , Olszewski, N. , Ori, N. , Eshed, Y. and Weiss, D. (2005) Cross talk between gibberellin and cytokinin: the Arabidopsis GA response inhibitor SPINDLY plays a positive role in cytokinin signaling. Plant Cell, 17, 92–102. 10.1105/tpc.104.028472. 15608330 PMC544492

[tpj14202-bib-0018] Gregis, V. , Sessa, A. , Dorca‐Fornell, C. and Kater, M.M. (2009) The Arabidopsis floral meristem identity genes *AP1, AGL24* and *SVP* directly repress class B and C floral homeotic genes. Plant J. 60, 626–637. 10.1111/j.1365-313X.2009.03985.x. 19656343

[tpj14202-bib-0107] Hackbusch, J. , Richter, K. , Müller, J. , Salamini, F. and Uhrig, J.F. (2005) A central role of Arabidopsis thaliana ovate family proteins in networking and subcellular localization of 3‐aa loop extension homeodomain proteins. Proc. Natl. Acad. Sci. U S A, 102, 4908–4912. 10.1073/pnas.0501181102. 15781858 PMC555730

[tpj14202-bib-0019] Harkess, A. , Mercati, F. , Shan, H.Y. , Sunseri, F. , Falavigna, A. and Leebens‐Mack, J. (2015) Sex‐biased gene expression in dioecious garden asparagus (*Asparagus officinalis*). New Phytol. 207, 883–892. 10.1111/nph.13389. 25817071

[tpj14202-bib-0020] Harkess, A. , Zhou, J. , Xu, C. ***et al.*** (2017) The asparagus genome sheds light on the origin and evolution of a young y chromosome. Nat. Commun. 8, 1279 10.1038/s41467-017-01064-8. 29093472 PMC5665984

[tpj14202-bib-0021] Hartmann, U. , Höhmann, S. , Nettesheim, K. , Wisman, E. , Saedler, H. and Huijser, P. (2000) Molecular cloning of *SVP*: a negative regulator of the floral transition in Arabidopsis. Plant J. 21, 351–360. 10.1046/j.1365-313x.2000.00682.x 10758486

[tpj14202-bib-0022] Henry, I.M. , Akagi, T. , Tao, R. and Comai, L. (2018) One hundred ways to invent the sexes: theoretical and observed paths to dioecy in plants. Annu. Rev. Plant Biol. 69, 553–575. 10.1146/annurev-arplant-042817-040615. 29719167

[tpj14202-bib-0023] Hu, Z. , Mellor, J. and DeLisi, C. (2004) Analyzing networks with VisANT. Curr. Protoc. Bioinformatics, 8, 8.8 10.1002/0471250953.bi0808s08. 18428738

[tpj14202-bib-0024] Hutchison, C.E. and Kieber, J.J. (2002) Cytokinin signaling in Arabidopsis. Plant Cell, 14, S47–S59 10.1105/tpc.010444. 12045269 PMC151247

[tpj14202-bib-0025] Itkin, M. , Heinig, U. , Tzfadia, O. ***et al.*** (2013) Biosynthesis of antinutritional alkaloids in solanaceous crops is mediated by clustered genes. Science, 341, 175–179. 10.1126/science.1240230. 23788733

[tpj14202-bib-0108] Jasinski, S. , Piazza, P. , Craft, J. , Hay, A. , Woolley, L. , Rieu, I. , Phillips, A. , Hedden, P. and Tsiantis, M. (2005) KNOX action in Arabidopsis is mediated by coordinate regulation of cytokinin and gibberellin activities. Curr Biol, 15, 1560–1565. 10.1016/j.cub.2005.07.023. 16139211

[tpj14202-bib-0115] Kawamoto, T. (2003) Use of a new adhesive film for the preparation of multi‐purpose fresh‐frozen sections from hard tissues, whole‐animals, insects and plants. Arch. Histol. Cytol., 66, 123–143. 10.1679/aohc.66.123. 12846553

[tpj14202-bib-0027] Kazama, Y. , Ishii, K. , Aonuma, W. ***et al.*** (2016) A new physical mapping approach refines the sex‐determining gene positions on the *Silene latifolia* Y‐chromosome. Sci. Rep. 6, 18917 10.1038/srep18917. 26742857 PMC4705512

[tpj14202-bib-0028] Khan, A. , Fornes, O. , Stigliani, A. ***et al.*** (2018) JASPAR 2018: update of the open‐access database of transcription factor binding profiles and its web framework. Nucleic Acids Res. 46(D1), D260–D266. 10.1093/nar/gkx1126. 29140473 PMC5753243

[tpj14202-bib-0029] Koizumi, A. , Yamanaka, K. , Nishihara, K. , Kazama, Y. , Abe, T. and Kawano, S. (2010) Two separate pathways including *SlCLV1*,* SlSTM* and *SlCUC* that control carpel development in a bisexual mutant of silene latifolia. Plant Cell Physiol. 51, 282–293. 10.1093/pcp/pcp187. 20064843

[tpj14202-bib-0030] Köllmer, I. , Novák, O. , Strnad, M. , Schmülling, T. and Werner, T. (2014) Overexpression of the cytosolic cytokinin oxidase/dehydrogenase (CKX7) from Arabidopsis causes specific changes in root growth and xylem differentiation. Plant J. 78, 359–371. 10.1111/tpj.12477. 24528491

[tpj14202-bib-0031] Langfelder, P. and Horvath, S. (2008) WGCNA: an R package for weighted correlation network analysis. BMC Bioinformatics, 9, 559 10.1186/1471-2105-9-559. 19114008 PMC2631488

[tpj14202-bib-0032] Larue, N.C. , Sullivan, A.M. and Di Stilio, V.S. (2013) Functional recapitulation of transitions in sexual systems by homeosis during the evolution of dioecy in *Thalictrum* . Front. Plant Sci. 4, 487 10.3389/fpls.2013.00487. 24348491 PMC3842162

[tpj14202-bib-0033] Lebel‐Hardenack, S. and Grant, S.R. (1997) Genetics of sex determination in flowering plants. Trends Plant Sci. 2, 130–136. 10.1016/S1360-1385(97)01012-1.

[tpj14202-bib-0034] Li, H. and Durbin, R. (2009) Fast and accurate short read alignment with Burrows‐Wheeler transform. Bioinformatics, 25, 1754–1760. 10.1093/bioinformatics/btp324. 19451168 PMC2705234

[tpj14202-bib-0035] Li, Y. , Pearl, S.A. and Jackson, S.A. (2015) Gene networks in plant biology: approaches in reconstruction and analysis. Trends Plant Sci. 2, 664–675. 10.1016/j.tplants.2015.06.013. 26440435

[tpj14202-bib-0036] Liseron‐Monfils, C. and Ware, D. (2015) Revealing gene regulation and associations through biological networks. Curr. Plant Biol. 4, 30–39. 10.1016/j.cpb.2015.11.001.

[tpj14202-bib-0037] Liu, Z. , Moore, P.H. , Ma, H. ***et al.*** (2004) A primitive Y chromosome in papaya marks incipient sex chromosome evolution. Nature, 427, 348–352. 10.1038/nature02228. 14737167

[tpj14202-bib-0038] Liu, C. , Chen, H. , Er, H.L. , Soo, H.M. , Kumar, P.P. , Han, J.H. , Liou, Y.C. and Yu, H. (2008) Direct interaction of *AGL24* and *SOC1* integrates flowering signals in Arabidopsis. Development, 135, 1481–1491. 10.1242/dev.020255. 18339670

[tpj14202-bib-0039] Machanick, P. and Bailey, T.L. (2011) MEME‐ChIP: motif analysis of large DNA datasets. Bioinformatics, 25, 1696–1697. 10.1093/bioinformatics/btr189. PMC3106185 21486936

[tpj14202-bib-0040] Marsch‐Martínez, N. , Ramos‐Cruz, D. , Reyes‐Olalde, I.J. , Lozano‐Sotomayor, P. , Zúñiga‐Mayo, V.M. and DeFolter, S. (2012) The role of cytokinin during Arabidopsis gynoecia and fruit morphogenesis and patterning. Plant J. 72, 222–234. 10.1111/j.1365-313X.2012.05062.x. 22640521

[tpj14202-bib-0041] McCarthy, D.J. , Chen, Y. and Smyth, G.K. (2012) Differential expression analysis of multifactor RNA‐Seq experiments with respect to biological variation. Nucleic Acids Res. 40, 4288–4297. 10.1093/nar/gks042. 22287627 PMC3378882

[tpj14202-bib-0042] Ming, R. , Bendahmane, A. and Renner, S.S. (2011) Sex chromosomes in land plants. Annu. Rev. Plant Biol. 62, 485–514. 10.1146/annurev-arplant-042110-103914. 21526970

[tpj14202-bib-0043] Mizukami, Y. and Ma, H. (1997) Determination of Arabidopsis floral meristem identity by *AGAMOUS* . Plant Cell, 9, 393–408. 10.1105/tpc.9.3.393. 9090883 PMC156926

[tpj14202-bib-0044] Müller, B. and Sheen, J. (2007) Advances in cytokinin signaling. Science, 318, 68–6. 17916725 10.1126/science.1145461

[tpj14202-bib-0045] Muyle, A. , Shearn, R. and Marais, G.A. (2017) The evolution of sex chromosomes and dosage compensation in plants. Genome Biol. Evol. 9(3), 627–645. 10.1093/gbe/evw282. 28391324 PMC5629387

[tpj14202-bib-0118] Nemhauser, J.L. , Feldman, L.J. and Zambryski, P.C. (2000) Auxin and ETTIN in Arabidopsis gynoecium morphogenesis. Development, 127, 3877‐3888. http://dev.biologists.org/content/127/18/3877.long 10952886 10.1242/dev.127.18.3877

[tpj14202-bib-0113] O'Malley, R.C. , Huang, S.C. , Song, L. , Lewsey, M.G. , Bartlett, A. , Nery, J.R. , Galli, M. , Gallavotti, A. and Ecker, J.R. (2016) Cistrome and epicistrome features shape the regulatory DNA landscape. Cell, 165, 1280–1292. 10.1016/j.cell.2016.04.038. 27203113 PMC4907330

[tpj14202-bib-0046] Pagnussat, G.C. , Yu, H.J. and Sundaresan, V. (2007) Cell‐fate switch of synergid to egg cell in Arabidopsis eostre mutant embryo sacs arises from misexpression of the BEL1‐Like Homeodomain Gene *BLH1* . Plant Cell, 19, 3578–3592. 10.1105/tpc.107.054890. 18055603 PMC2174879

[tpj14202-bib-0047] Paponov, I.A. , Paponov, M. , Teale, W. , Menges, M. , Chakrabortee, S. , Murray, J.A.H. and Palme, K. (2008) Comprehensive transcriptome analysis of auxin responses in Arabidopsis. Mol. Plant, 1, 321–337. 10.1093/mp/ssm021. 19825543

[tpj14202-bib-0048] Pfent, C. , Pobursky, K.J. , Sather, D.N. and Golenberg, E.M. (2005) Characterization of *SpAPETALA3* and *SpPISTILLATA*, B class floral identity genes in *Spinacia oleracea*, and their relationship to sexual dimorphism. Dev. Genes. Evol. 215, 132–142. 10.1007/s00427-004-0459-4. 15660251

[tpj14202-bib-0049] Renner, S.S. (2014) The relative and absolute frequencies of angiosperm sexual systems: dioecy, monoecy, gynodioecy, and an updated online database. Am. J. Bot. 101, 1588–1596. 10.3732/ajb.1400196. 25326608

[tpj14202-bib-0050] Rijpkema, A.S. , Vandenbussche, M. , Koes, R. , Heijmans, K. and Gerats, T. (2010) Variations on a theme: changes in the floral ABCs in angiosperms. Semin. Cell Dev. Biol. 21, 100–107. 10.1016/j.semcdb.2009.11.002. 19932760

[tpj14202-bib-0051] Robinson, M.D. , McCarthy, D.J. and Smyth, G.K. (2010) edgeR: a Bioconductor package for differential expression analysis of digital gene expression data. Bioinformatics, 26, 139–140. 10.1093/bioinformatics/btp616. 19910308 PMC2796818

[tpj14202-bib-0116] Rybel, B.D. , Berg, W. , Lokerse, A. , Liao, C.H. , Mourik, H. , Möller, B. , Peris, C.L. and Weijers, D. (2011) A versatile set of ligation‐independent cloning vectors for functional studies in plants. Plant Physiol., 156, 1292–1299. 10.1104/pp.111.177337. 21562332 PMC3135924

[tpj14202-bib-0052] Sakamoto, T. , Kamiya, N. , Ueguchi‐Tanaka, M. , Iwahori, S. and Matsuoka, M. (2001) KNOX homeodomain protein directly suppresses the expression of a gibberellin biosynthetic gene in the tobacco shoot apical meristem. Genes Dev. 15, 581–590. http://www.genesdev.org/cgi/doi/10.1101/gad.867901. 11238378 10.1101/gad.867901 PMC312643

[tpj14202-bib-0053] Sather, D.N. , Maja, J. and Golenberg, M. (2010) Functional analysis of B and C class floral organ genes in spinach demonstrates their role in sexual dimorphism. BMC Plant Biol. 10, 46 10.1186/1471-2229-10-46. 20226063 PMC2923521

[tpj14202-bib-0054] Scofield, S. , Dewitte, W. and Murray, J.A.H. (2007) The *KNOX* gene *SHOOT MERISTEMLESS* is required for the development of reproductive meristematic tissues in Arabidopsis. Plant J. 50, 767–781. 10.1111/j.1365-313X.2007.03095.x. 17461793

[tpj14202-bib-0055] Serin, E.A.R. , Nijveen, H. , Hilhorst, H.W.M. and Ligterink, W. (2016) Learning from co‐expression networks: possibilities and challenges. Front. Plant Sci. 7, 444 10.3389/fpls.2016.00444. 27092161 PMC4825623

[tpj14202-bib-0056] Smyth, D.R. , Bowman, J.L. and Meyerowitz, E.M. (1990) Early flower development in Arabidopsis. Plant Cell, 2, 755–767. 10.1105/tpc.2.8.755. 2152125 PMC159928

[tpj14202-bib-0057] Sobral, R. and Costa, M.M.R. (2017) Role of floral organ identity genes in the development of unisexual flowers of *Quercus suber* L. Sci. Rep. 7, 10368 10.1038/s41598-017-10732-0. 28871195 PMC5583232

[tpj14202-bib-0058] Tian, T. , Liu, Y. , Yan, H. , You, Q. , Yi, X. , Du, Z. , Xu, W. and Su, Z. (2017) AgriGO v2.0: a GO analysis toolkit for the agricultural community, 2017 update. Nucleic Acids Res. 45, W122–W129. 10.1093/nar/gkx382. 28472432 PMC5793732

[tpj14202-bib-0059] Tsaballa, A. , Pasentsis, K. , Darzentas, N. and Tsaftaris, A.S. (2011) Multiple evidence for the role of an ovate‐like gene in determining fruit shape in pepper. BMC Plant Biol. 11, 46 10.1186/1471-2229-11-46. 21401913 PMC3069956

[tpj14202-bib-0119] Ueda, A. , Li, P. , Feng, Y. ***et al.*** (2008) The Arabidopsis thaliana carboxyl‐terminal domain phosphatase‐like 2 regulates plant growth, stress and auxin responses. Plant Mol. Biol., 67, 683‐697. 10.1007/s11103-008-9348-y. 18506580

[tpj14202-bib-0060] Wagner, D. (2008) Flower morphogenesis: timing is key. Dev. Cell, 16, 621–622. 10.1016/j.devcel.2009.05.005. 19460335

[tpj14202-bib-0061] Wang, S. , Chang, Y. , Guo, J. , Zeng, Q. , Ellis, B. and Chen, J. (2011) Arabidopsis ovate family proteins, a novel transcriptional repressor family, control multiple aspects of plant growth and development. PLoS One, 6(8), e23896 10.1371/journal.pone.0023896. 21886836 PMC3160338

[tpj14202-bib-0062] Wang, J. , Na, J.K. , Yu, Q. ***et al.*** (2012) Sequencing papaya X and Yh chromosomes reveals molecular basis of incipient sex chromosome evolution. Proc. Natl. Acad. Sci. USA, 109, 13710–13715. 10.1073/pnas.1207833109. 22869747 PMC3427123

[tpj14202-bib-0063] Wang, Z. , Jiao, Z. , Xu, P. , Chen, L. , Ai, J. , Liu, X. and Yang, Y. (2013) Bisexual flower ontogeny after chemical induction and berry characteristics evaluation in male *Vitis amurensis* Rupr. Sci. Hortic. 162, 11–19. 10.1016/j.scienta.2013.07.038.

[tpj14202-bib-0064] Wang, S. , Chang, Y. and Ellis, B. (2016) Overview of *OVATE FAMILY PROTEINS*, a novel class of plant‐specific growth regulators. Front. Plant Sci. 7, 417 10.3389/fpls.2016.00417. 27065353 PMC4814488

[tpj14202-bib-0065] Weigel, D. and Meyerowitz, E.M. (1994) The ABCs of floral homeotic genes. Cell, 78, 203–209. 10.1016/0092-8674(94)90291-7. 7913881

[tpj14202-bib-0066] Werner, T. and Schmülling, T. (2009) Cytokinin action in plant development. Curr. Opin. Plant Biol. 12, 527–538. 10.1016/j.pbi.2009.07.002. 19740698

[tpj14202-bib-0067] Wickham, H. (2009) Ggplot2: elegant graphics for data analysis Springer‐Verlag, New York ISBN 978‐3‐319‐24277‐4. http://ggplot2.org.

[tpj14202-bib-0068] Yanai, O. , Shani, E. , Dolezal, K. , Tarkowski, P. , Sablowski, R. , Sandberg, G. , Samach, A. and Ori, N. (2005) *Arabidopsis* KNOXI proteins activate cytokinin biosynthesis. Curr. Biol. 15, 1566–1571. 10.1016/j.cub.2005.07.060. 16139212

[tpj14202-bib-0069] Yanofsky, M.F. , Ma, H. , Bowman, J.L. , Drews, G.N. , Feldmann, K.A. and Meyerowitz, E.M. (1990) The protein encoded by the Arabidopsis homeotic gene agamous resembles transcription factors. Nature, 346, 35–39. 10.1038/346035a0. 1973265

[tpj14202-bib-0105] Yang, Y. , Hammes, U.Z. , Taylor, G.G. , Schachtman, D.P. and Nielsen, E. (2006) High‐affinity auxin transporter by the AUX1 influx carrier protein. Curr. Biol., 16, 1123–1127. 10.1016/j.cub.2006.04.029. 16677815

[tpj14202-bib-0102] Yanhui, C. , Xiaoyuan, Y. , Kun , Meihua, L. ***et al.*** (2006) Arabidopsis: expression analysis and phylogenetic comparison with the rice MYB family. Plant Mol. Biol., 60, 107–124. 10.1007/s11103-005-2910-y. 16463103

[tpj14202-bib-0070] Yonemori, K. , Sugiura, A. , Tanaka, K. and Kameda, K. (1993) Floral ontogeny and sex determination in monoecious‐type persimmons. J. Am. Soc. Hortic. Sci. 118, 293–297. http://journal.ashspublications.org/content/118/2/293.

[tpj14202-bib-0071] Zhang, Y. , Liu, T. , Meyer, C.A. ***et al.*** (2008) Model‐based analysis of ChIP‐Seq (MACS). Genome Biol. 9, R137 10.1186/gb-2008-9-9-r137. 18798982 PMC2592715

[tpj14202-bib-0072] Zhang, Y. , Zhao, G. , Li, Y. , Mo, N. , Zhang, J. and Liang, Y. (2017) Transcriptomic analysis implies that GA regulates sex expression via ethylene‐dependent and ethylene‐independent pathways in cucumber (*Cucumis sativus* L.). Front. Plant Sci. 8, 10 10.3389/fpls.2017.00010. 28154572 PMC5243814

[tpj14202-bib-0111] Zúñiga‐Mayo, V.M. , Reyes‐Olalde, J.I. , Marsch‐Martinez, N. and Folter, S. (2014) Cytokinin treatments affect the apical‐basal patterning of the Arabidopsis gynoecium and resemble the effects of polar auxin transport inhibition. Front Plant Sci., 5, 191 10.3389/fpls.2014.00191. 24860582 PMC4030163

