# Supplementary material for: Gene networks orchestrated by MeGI: a single‐factor mechanism underlying sex determination in persimmon
Source: Plant J. 2019 Feb 14;98(1):97–111. doi: 10.1111/tpj.14202 (PMC6850717; doi:10.1111/tpj.14202)
Supplement: Supplementary file 1 — Figure S1. Morphological characterization of stage 1 and stage 3. Figure S2. Visualization of the male module network. Figure S3. Pearson correlation matrix in the MeGI‐SVP‐SOC1 and PI/AG. Figure S4. Pearson correlation matrix in the whole genes between the female and male modules. Figure S5. Organ‐specificity in expression of the MeGI and the downstream candidates. [file TPJ-98-97-s001.docx]

**
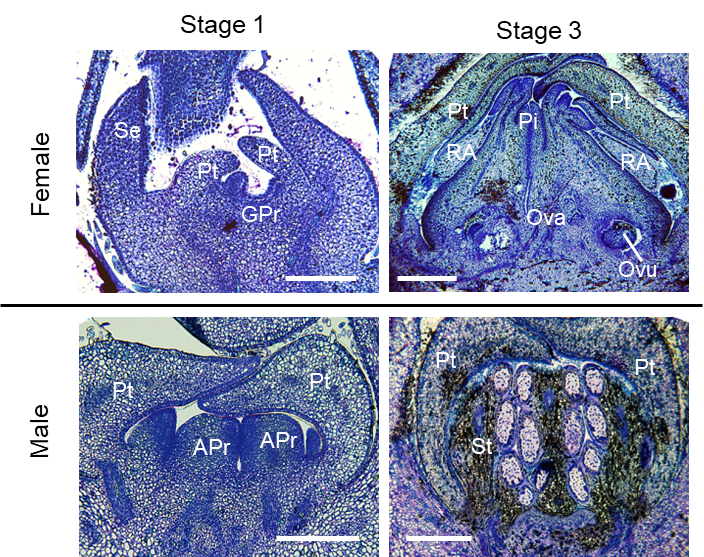
**

**Supporting Figure 1. Morphological characterization of stage 1 and stage 3.**

We defined 4 stages according to morphology and differentiation of gynoecium/androecium in male and female developing flowers. Cross sections of male and female flowers at stage 1and stage 3 were shown. The samples were stained with toluidine blue. Se: sepal, Pt: petal, GPr: gynoecium primordia, Apr: androecium primordia, RA: rudimentary anther, Pi: pistil, Ova: ovary, Ovu: ovule, St: stamen. At stage 1, location of gynoecium primordia (or rudimentary androecium primordia) of female flowers are still unclear, whereas male flowers already show a little more developed androecium primordia, which is consistent with previous observation (Akagi et al. 2016). At stage 3, distinct gynoecium constituted of pistial and ovary, and rudimentary anthers was observed in female flowers.

**
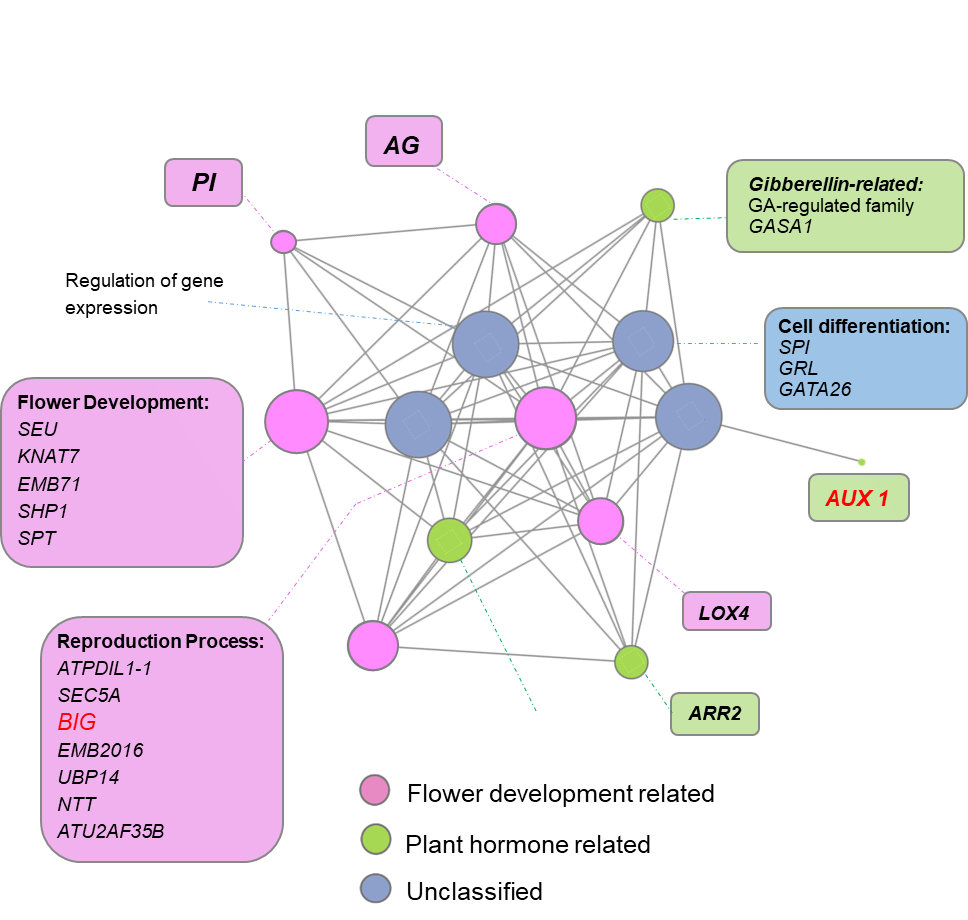
**

**Supporting Figure 2. Visualization of the male module network.**

This network is corresponded to the M4 module given in Figure 3A-B. The genes are clustered by their putative functions and given in different nodes. The gene clusters putatively related to plant hormone and flower organ development were shown in green and pink circles, respectively. The genes putatively related to auxin transportation, *AUX1* and *BIG*, were highlighted in red. The genes annotated with the function not representing any of them were given in blue (see Supporting Dataset S2). The size of the nodes reflects the number of edge connected to other nodes.


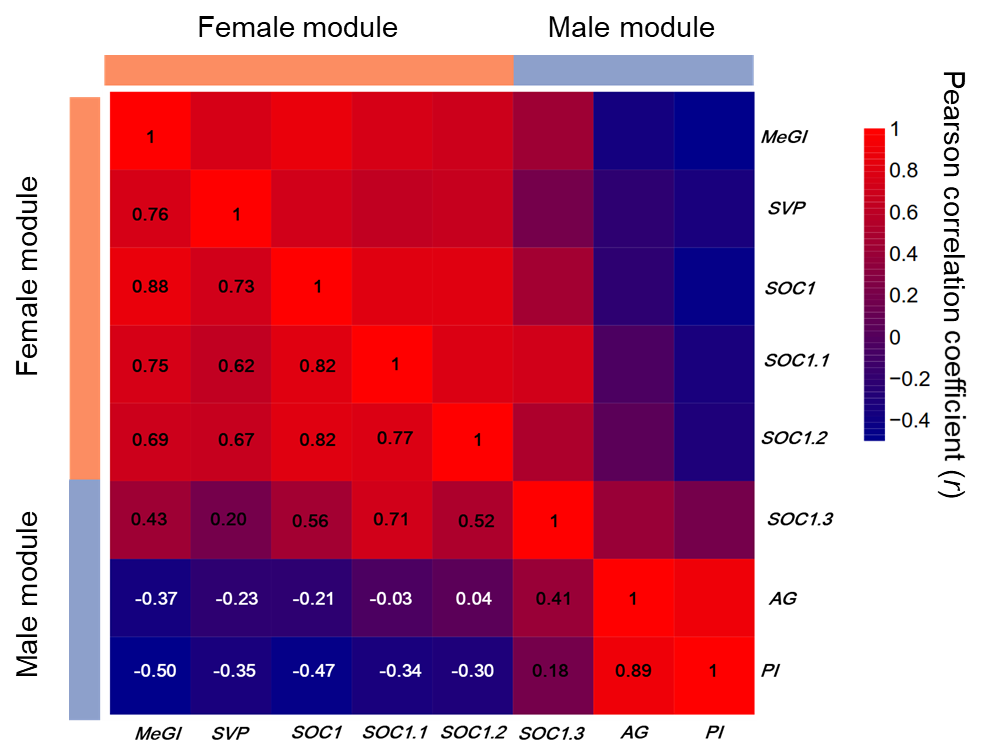


**Supporting Figure 3. Pearson correlation matrix in the *MeGI-SVP-SOC1* and *PI/AG.***

Pairwise correlation coefficient using the expression patterns in stage 1 were given in *MeGI*, *SVP*, *SOC1*, *AG* and *PI*. *SVP* and *SOC1*s tended to show significant negative correlation against *AG* and *PI*, which implied potential connections of the female and male modules. For *SOC1*, there are some paralogs and/or alleles annotated with the same *SOC1* as the highest hit, in the genome sequences of *D. lotus*. In this study, we mainly focused on Dlo_pri0359F.1_g00240.1 (see Supporting Dataset S1 and S2), simply annotated as “*SOC1*”. The alleles/paralogs (*SOC1.1* and *1.2*) showed similar expression patterns and nested to the female module, except *SOC1.3*.


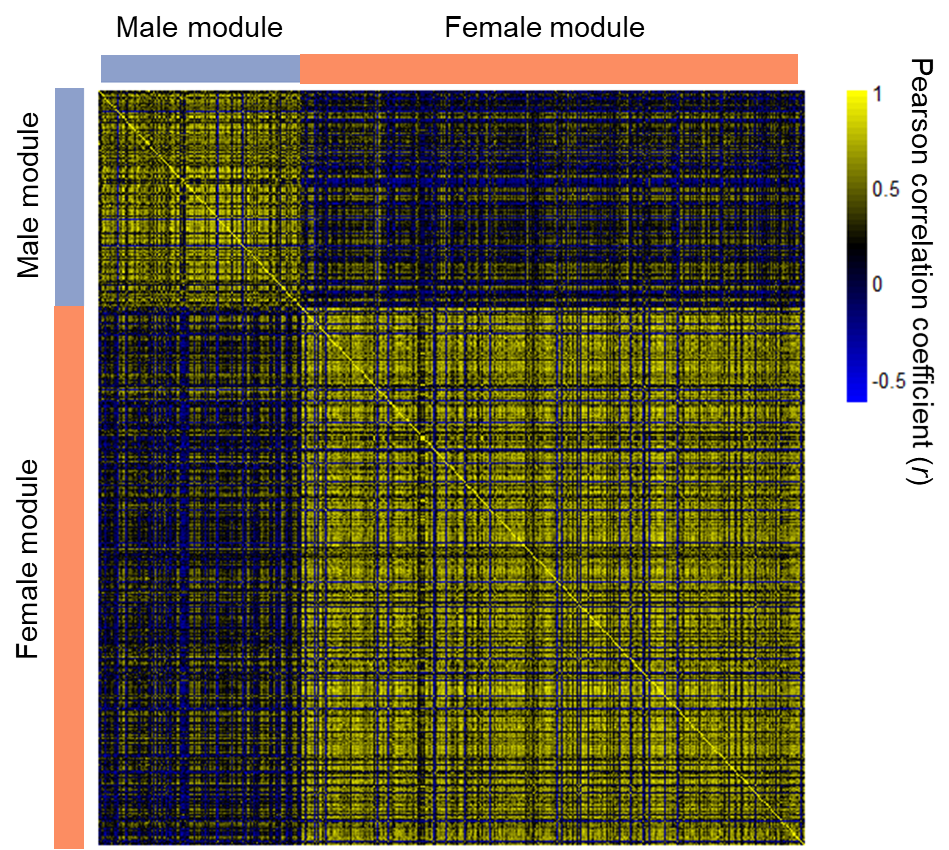


**Supporting Figure 4. Pearson correlation matrix in the whole genes between the female and male modules.**

Pairwise correlation coefficients across the genes nested to female and male modules, using the expression patterns in stage 1 and stage 3. The female and male modules tended to show negative correlation, suggesting their potential connections in the gene functions.


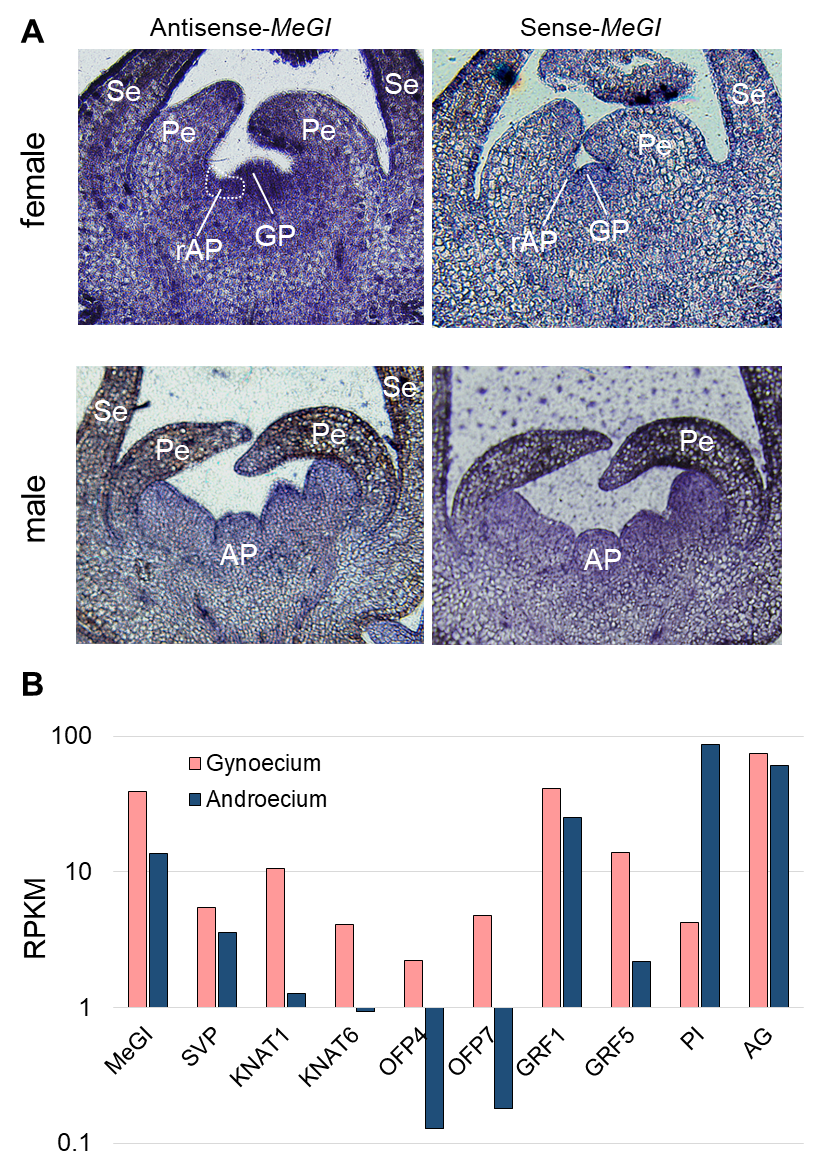


**Supporting Figure 5. Organ-specificity in expression of the *MeGI* and the downstream candidates.**

**A,** *in situ* RNA hybridization using antisense-*MeGI* and sense-*MeGI* sequences as the probes, in stage 1. In female flowers, strong *MeGI* signals were detected mainly in meristematic region including gynoecium primordia (GP) and rudimentary androecium primordia (rAP). The substantial *MeGI* signal was specific to female flowers, presumably due to the lack of *MeGI* expression in male flowers (Akagi et al. 2016). **B,** Normalized expression levels (RPKM) of *MeGI*, the genes putatively under the direct control of *MeGI* in the female module and *AG* and *PI*, in the mRNA-Seq analysis for gynoecium and androecium in stage 2. *MeGI*, *SVP*, *GRF*s, and *AG* showed no substantial bias in expression levels between gynoecium and androecium (< 5-fold change). On the other hand, *KNAT1*, *KNAT6*, *OFP4*, and *OFP7* showed gynoecium-biased, and *PI* showed androecium-biased expression. Of them, *KNAT6*, *OFP4*, and *OFP7* were nearly under the detection in androecium (RPKM < 1).
