# Supplementary material for: Gene networks orchestrated by MeGI: a single‐factor mechanism underlying sex determination in persimmon
Source: Plant J. 2019 Feb 14;98(1):97–111. doi: 10.1111/tpj.14202 (PMC6850717; doi:10.1111/tpj.14202)
Supplement: Supplementary file 2 — Table S1. List of plant materials. Table S2. List of the representative DEGs putatively related to androecium, gynoecium, and meristem development in stage 1 (a) and stage 3 (b). Table S3. Genes directly connected to MeGI in the female module. Table S4. Phenotypes of the MeGI‐overexpressed and control transgenic lines. Table S5. Enriched GO terms in the DEGs between the MeGI‐overexpressed and control in Arabidopsis. [file TPJ-98-97-s002.docx]

**Supporting Table S1**: List of plant materials

**Supporting Table S2** List of the representative DEGs putatively related to androecium, gynoecium, and meristem development in stage 1 (A) and stage 3 (B). The detail information of the DEGs were given in Supporting Dataset S1.

**A**

^a^Gene names used in this study, or information of the alternative alleles were given.

**B**

**Continued**

**Supporting Table S3**: Genes directly connected to *MeGI* in the female module

**Supporting Table S4**: Phenotypes of the *MeGI*-overexpressed and control transgenic lines

^a^ ++: Flowers with rudimentary anthers, +: Flowers with partially deficient anthers (Akagi et al. 2014).

^b^ ++: severe dwarfism with leaf serration, +: weak dwarfism

^c^ expression levels were semi-quantitatively assessed by RT-PCR

^d^ lines used for mRNA-Seq, given in Figure 5, Supporting Table 5, and Supporting Dataset S3.

**Supporting Table S5**: Enriched GO terms in the DEGs between the *MeGI*-overexpressed and control in Arabidopsis
